# Supplementary material for: Technical efficiency of national HIV/AIDS spending in 78 countries between 2010 and 2018: A data envelopment analysis
Source: PLOS Glob Public Health. 2022 Aug 1;2(8):e0000463. doi: 10.1371/journal.pgph.0000463 (PMC10022340; doi:10.1371/journal.pgph.0000463)
Supplement: S1 Text — (DOCX) [file pgph.0000463.s001.docx]

# SUPPLEMENTARY MATERIAL

# Technical efficiency of national HIV/AIDS spending in 78 countries between 2010 and 2018: a data envelopment analysis

Kasim ALLEL, Gerard Joseph ABOU JAOUDE, Charles BIRUNGI, Tom PALMER, Jolene SKORDIS, Hassan HAGHPARAST-BIDGOLI

Table of contents

Section A: Technical appendix……………………………………………………………..2

Data envelopment and technical efficiency…………….…………………………….2

DEA analysis: Simar and Wilson two-stage approach……………………………….3

Multivariate regression for data imputation and testing……………………………...6

Section B: Additional analyses including figures and tables…………………………...…8

References…………………………………………………………………………………....42

**Section A: Technical appendix**

**Data Envelopment Analysis methodology and Simar and Wilson two-stage approach**

Data envelopment and technical efficiency

Technical efficiency is described as the performance level of each Decision-Making Unit (DMU) relative to an efficient technology (hypothetical scenario) constituted by a frontier function (1, 2). We used the Data envelopment Analysis (DEA) for determining this frontier in the current paper and to evaluate the efficiency of each DMU. We chose this non-parametric method due to its no re-strictive hypothesis on the data generating process which require very few assumptions. We use the double bootstrap method developed by Simar & Wilson to measure the technical efficiency of HIV spending in our sampled countries (DMUs), and to assess the main independent variables associated to efficiency. The stage approach is comprised of a first stage consisting of the estimation of efficiency scores for each DMU, while the second stage aims to regress those efficiency scores accounting for external independent variables using a truncated model. One of the main disadvantages of the DEA analyses is that they suffer from serial correlation (presence of endogeneity due to measurement error in the scores); therefore, we used the Simar & Wilson bootstrap approach to correct for measurement error and serial correlation.

Briefly, the DEA method solves the optimisation problem to estimate technical efficiency that is as follows:

max ($\frac{\sum_{s=1}^{S} u_{s}y_{s0}}{\sum_{m=1}^{M} v_{m}x_{m0}}$) ; subject to $\frac{\sum_{s=1}^{S} u_{s}y_{s0}}{\sum_{m=1}^{M} v_{m}x_{m0}}\leq1, i=1,..,I$

where:

𝑦𝑠0 is the quantity of outputs for DMU0
𝑢𝑠 is the weight of output s and 𝑢𝑠 > 0
𝑥𝑚0 is the quantity of output m for DMU0
𝑣𝑚 is the weight of output s and 𝑣𝑚 > 0
s=1,..,S, m=1,..,M

As for an output-oriented approach, the sum of the inputs must be held constant and the denominator is equal to 1, so the optimization problem is rewritten as follows:

max ($\frac{\sum_{s=1}^{S} u_{s}y_{s0}}{1}$) ;

subject to

$$\sum_{m=1}^{M} v_{m}x_{m0}=1, i=1,..,I$$

$\sum_{r=1}^{S} ury\mathrm{rj}$ −$\sum_{i=1}^{M} v_{m}x_{mi}$ ≤0
 𝑖=1,...𝑛 ; 𝑢𝑟,𝑣𝑖 ≥0

The sum of all outputs cannot exceed the sum of all inputs and the weight for each variable has to be positive; therefore, it secures that all inputs/outputs are part of the solution (efficiency scores)

We can rewrite the maximisation but using a set of linear equations as follows:

𝑀𝑎𝑥𝑣,𝑢 𝜃_0_ = 𝑢𝑦𝑖

subject to:

𝑣𝑥𝑖 = 1 −𝑣𝑋 + 𝑢𝑌 ≤ 0 𝑢, 𝑣 ≥ 0

𝑥𝑖 and 𝑦𝑖 represent input and output vectors for all ‘i’ DMUs; 𝑢,𝑣 are row vectors for input and output weights, and X, Y are input and output matrices representing data for all I DMUs. 𝜃 is a scalar and 𝜃 ≤ 1, representing the level of technical efficiency with 1 being fully efficient and 0 null efficiency, compared to the rest.

DEA analysis: Simar and Wilson two-stage approach

Simar and Wilson introduced a type of DEA analysis that is comprised of two different parts (3). In the **first stage,** bootstrapped DEA scores are computed using an output or input-oriented approach with multiple inputs and outputs, which follows the classic DEA optimization problem as described above. For this study purposes, we used an output-oriented approach with multiple inputs. In the **second stage**, a bootstrapped truncated regression is used by regressing the bias-corrected scores on different independent variables that were detailed in the manuscript.

The two stages of the Simar and Wilson can be translated into a 7 steps procedure that is described hereinafter and follows their algorithm 2 (steps 1-4 are part of the first stage, whereas steps 5-7 are part of the second stage)(3, 4).

1. For steps 1 to 4, Simar and Wilson estimate the bias-corrected efficiency scores of the Shepard distance function $\hat{\hat{\theta}}$, by subtracting the bootstrap bias estimate $(\hat{\theta}$) from the original distance function estimate following the equation detailed below. $\hat{\theta}$ is estimated using the DEA approach (in this case the output-oriented maximisation process).

$\hat{\hat{\theta}}$= $\hat{\theta}-\overbrace{BIAS(\hat{\theta})}$

Truncated maximum likelihood is performed to regress those scores on a set of independent variables by employing bootstrap techniques (as for this study we used 1,000 repetitions from the truncated normal distribution of the estimated inefficiency scores). By employing this method, we estimate the true sampling distribution through the construction of small samples while resolving DEA for every DMU within the sample and repeating the process as many times as we get a good approximation of the distribution of the scores. Then, bias-corrected estimates are calculated.

1. For steps 5-7, Simar and Wilson regress the bias-corrected technical inefficiency scores over a set of exogenous covariates using a bootstrapped truncated regression. We used 3,000 iterations to obtain unbiased coefficients and 95% confidence intervals. As we mentioned inefficiency, the reciprocal values of technical efficiency were used in the regression model as the dependent variable, which means that our scores had to be converted after computing the method, as we detailed in methods. The reciprocal values (also called inefficiency or the reciprocal of the efficiency score with the range from one to infinity) are expressed by the following term: (1/$\hat{\hat{\theta}}$). For instance, we show the association of independent variables with (1/$\hat{\hat{\theta}}$) a negative value for Rule of Law was associated with a negative (1/$\hat{\hat{\theta}}$) value or ratio, which means that a higher efficiency score is observed, given by the $\hat{\hat{\theta}}$ parameter. This is translated into the following equation for the truncated regression that consists of adjusting the inefficiency estimates to independent variables (bias corrected).

(1/$\hat{\hat{\theta}}$) = β_0_ + β_1_; ...; β_n_ E_it_ + μ_it ;_

i= countries, t= time in years, n= n number of coefficients per “n” determinants or independent variables, $\hat{\hat{\theta}}$= technical efficiency score, β_0_ is a constant term, and μ is an error term, (1/$\hat{\hat{\theta}}$)= technical inefficiency scores, 1<(1/$\hat{\hat{\theta}}$)< ∞, whereas 0<$\hat{\hat{\theta}}$<1.

This is also specified on the RStudio package rDEA that we used for the analyses (<https://www.rdocumentation.org/packages/rDEA/versions/1.2-6/topics/dea.env.robust>).


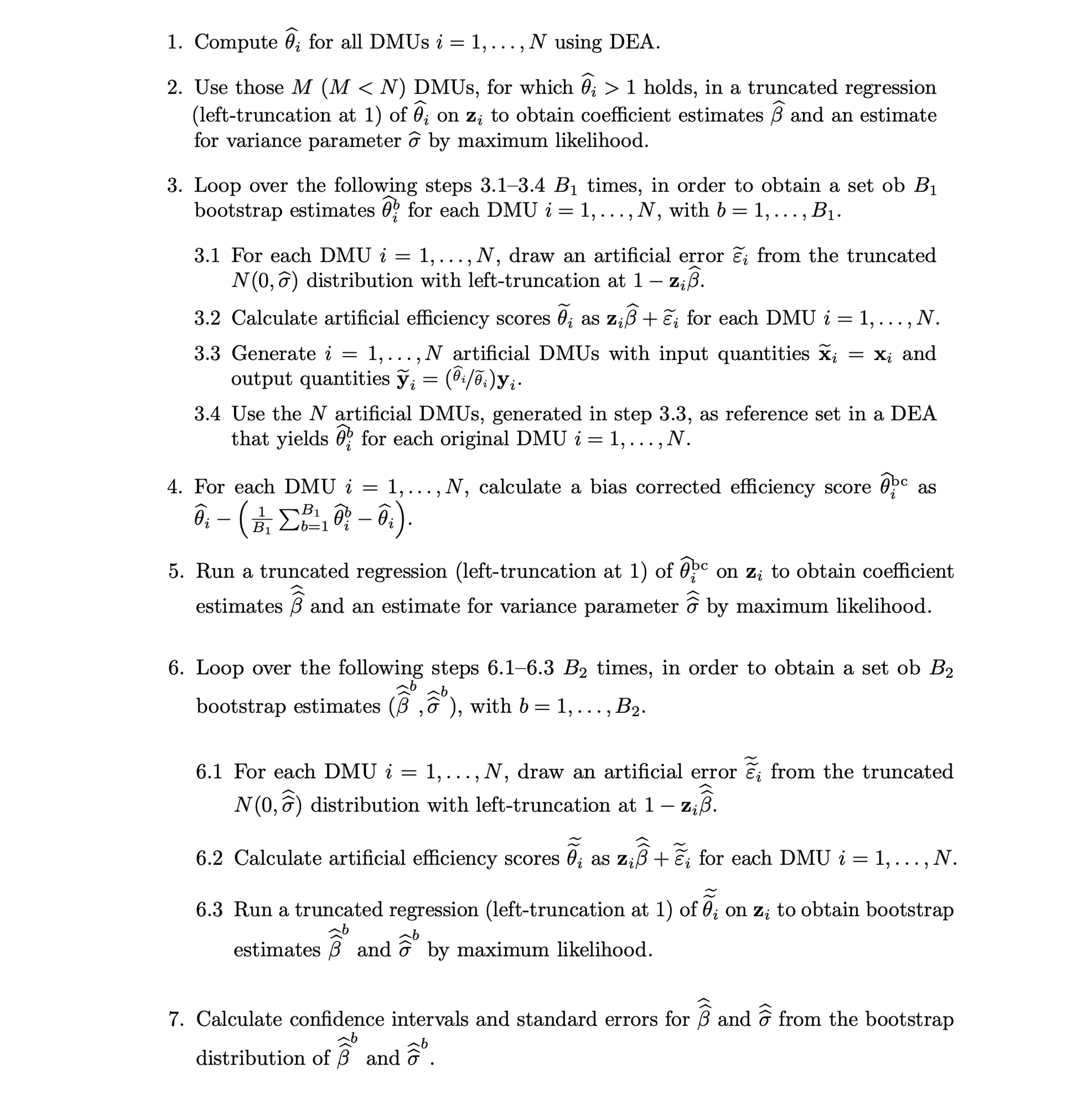
A full step to step procedure of the Simar and Wilson method (algorithm 2), previously adapted by Badunenko and Tauchmann (5), is showed below.

**Multivariate regression for data imputation and testing**

We considered the following multivariate normal regression for imputing missing values (6):

x_pij_ =Θ′z_qij_ +ε_ij,_ ; i=1,...,N, j= 2000,… 2018

where z_qij_ is a matrix of ‘q’ independent (complete) variables from observation “i” at time “j”.

Θ is a q × p matrix of regression coefficients, and ε_i_ is a p × 1 vector of random errors from a p-variate normal distribution. x_pij_ is a matrix of ‘p’ dependent (uncomplete) variables from observation “I” at time “j”. Matrix Z is comprised of Raw of Law, GDP, Government political stability, and Countries’ accountability.

As for the diagnostics, we employed descriptive statistics by using t-tests for the imputed and unimputed variables (see table below). Non meaningful statistical differences were found between imputed and original (not imputed) variables (see Table AA1 below).

Table A. Differences in descriptive statistics between the main variables included before and after imputation, and t-test results

|  | Mean | Std. error | SD | 95% CI | |
| --- | --- | --- | --- | --- | --- |
| **Antenatal Care Coverage** |  |  |  |  |  |
| Difference original - imputed value | 2.5E-08 | 6.56E-08 | 1.78E-06 | -1.04E-07 | 1.54E-07 |
| P-value { *Ha: mean(diff)*≠*0* } | 0.7 |  |  |  |  |
| **GNI per capita** |  |  |  |  |  |
| Difference original - imputed value | 0.00 | 9.64E-06 | 2.94E-04 | -2.92E-05 | 8.70E-06 |
| P-value { *Ha: mean(diff)*≠*0* } | 0.29 |  |  |  |  |
| **CHE as a %** |  |  |  |  |  |
| Difference original - imputed value | -2.50E-09 | 6.23E-09 | 1.68E-07 | -1.47E-08 | 9.73E-09 |
| P-value { *Ha: mean(diff)*≠*0* } | 0.69 |  |  |  |  |
| **CHEper person** |  |  |  |  |  |
| Difference original - imputed value | 8.32E-07 | 1.08E-06 | 2.93E-05 | -1.30E-06 | 2.96E-06 |
| P-value { *Ha: mean(diff)*≠*0* } | 0.44 |  |  |  |  |
| **HDI** |  |  |  |  |  |
| Difference original - imputed value | 8.30E-11 | 5.09E-10 | 1.56E-08 | -9.17E-10 | 1.08E-09 |
| P-value { *Ha: mean(diff)*≠*0* } | 0.87 |  |  |  |  |
| **HIV prevalence** |  |  |  |  |  |
| Difference original - imputed value | -1.41E-09 | 4.41E-09 | 1.29E-07 | -1.01E-08 | 7.25E-09 |
| P-value { *Ha: mean(diff)*≠*0* } | 0.75 |  |  |  |  |
| **Population per km2** |  |  |  |  |  |
| Difference original - imputed value | -2.17E-07 | 4.97E-07 | 1.52E-05 | -1.19E-06 | 7.59E-07 |
| P-value { *Ha: mean(diff)*≠*0* } | 0.66 |  |  |  |  |

Notes: HIV spending per person, Rule of Law were not imputed and so they were not included.

Also, we found that the variation of our variables was augmented (as expected and to account for uncertainty; see Table AA2 below). The relative efficiency of the imputation (how well the true population parameters are estimated) was nearly 1, which indicates similarity between the estimates and true population parameters.

Table B. Imputation diagnostics

|  | Imputation variance | | |  |  |  | Relative |
| --- | --- | --- | --- | --- | --- | --- | --- |
|  | Within | | Between | Total | RVI | FMI | efficiency |
| Antenatal Care Coverage | | 0.00 | 0.00 | 0.00 | 0.46 | 0.32 | **0.994** |
| Rule of Law | 0.71 | | 0.21 | 0.93 | 0.29 | 0.23 | **0.995** |
| GNI per capita | 0.00 | | 0.00 | 0.00 | 2.09 | 0.68 | **0.987** |
| CHE as a % of the GDP | 0.05 | | 0.01 | 0.06 | 0.29 | 0.23 | **0.996** |
| CHE per capita | 0.00 | | 0.00 | 0.00 | 1.74 | 0.64 | **0.987** |
| HDI | 39.74 | | 18.94 | 59.06 | 0.49 | 0.33 | **0.993** |
| HIV prevalence | 0.01 | | 0.00 | 0.01 | 0.27 | 0.22 | **0.996** |
| Population per km^2^ | 0.00 | | 0.00 | 0.00 | 31.94 | 0.97 | **0.981** |

Notes: RVI (Relative Increase in Variance), FMI (Fraction of Missing Information), RE (Relative Efficiency).

**Section B: Additional analyses including figures and tables**

**Table A.** Countries and number of observations per country (N=78)

| **Country** | **Code** | **Obs** | **Country** | **Code** | **Obs** | **Country** | **Code** | **Obs** |
| --- | --- | --- | --- | --- | --- | --- | --- | --- |
| Algeria | DZA | 3 | Eswatini | SWZ | 9 | Nepal | NPL | 6 |
| Angola | AGO | 9 | Ethiopia | ETH | 9 | Niger | NER | 8 |
| Argentina | ARG | 9 | Gabon | GAB | 9 | Nigeria | NGA | 9 |
| Armenia | ARM | 9 | Gambia | GMB | 9 | Papua New Guinea | PNG | 9 |
| Azerbaijan | AZE | 1 | Georgia | GEO | 9 | Paraguay | PRY | 9 |
| Belarus | BLR | 9 | Ghana | GHA | 9 | Peru | PER | 9 |
| Benin | BEN | 9 | Guatemala | GTM | 9 | Portugal | PRT | 9 |
| Bolivia (Plurinational State of) | BOL | 9 | Guinea-Bissau | GNB | 9 | Republic of Moldova | MDA | 9 |
| Botswana | BWA | 9 | Haiti | HTI | 9 | Romania | ROU | 9 |
| Burkina Faso | BFA | 9 | Honduras | HND | 9 | Rwanda | RWA | 9 |
| Burundi | BDI | 9 | Indonesia | IDN | 2 | Senegal | SEN | 9 |
| Cambodia | KHM | 9 | Iran (Islamic Republic of) | IRN | 9 | Sierra Leone | SLE | 9 |
| Cameroon | CMR | 9 | Kazakhstan | KAZ | 9 | South Africa | ZAF | 9 |
| Central African Republic | CAF | 9 | Kenya | KEN | 9 | Spain | ESP | 9 |
| Chad | TCD | 9 | Kyrgyzstan | KGZ | 9 | Sudan | SDN | 4 |
| Chile | CHL | 9 | Lao People's Democratic Republic | LAO | 9 | Suriname | SUR | 9 |
| Colombia | COL | 9 | Lesotho | LSO | 9 | Tajikistan | TJK | 9 |
| Costa Rica | CRI | 9 | Liberia | LBR | 9 | Thailand | THA | 9 |
| Cuba | CUB | 9 | Malawi | MWI | 9 | Togo | TGO | 9 |
| Côte d'Ivoire | CIV | 9 | Malaysia | MYS | 9 | Ukraine | UKR | 9 |
| Democratic Republic of the Congo | COD | 9 | Mali | MLI | 9 | Uzbekistan | UZB | 6 |
| Djibouti | DJI | 9 | Mauritania | MRT | 9 | Viet Nam | VNM | 9 |
| Dominican Republic | DOM | 9 | Mexico | MEX | 9 | Zambia | ZMB | 9 |
| Ecuador | ECU | 9 | Morocco | MAR | 1 | Zimbabwe | ZWE | 9 |
| El Salvador | SLV | 9 | Mozambique | MOZ | 9 |  |  |  |
| Equatorial Guinea | GNQ | 9 | Myanmar | MMR | 7 |  |  |  |
| Eritrea | ERI | 9 | Namibia | NAM | 9 |  |  |  |

*Notes*: Obs stands for the number of observations the country has.

**Table B.** Correlation table for the variables included in the main (core) multivariate model from Table 4

| Variables | Rule of Law | Antenatal Care Coverage | GNI per capita in USD | CHE as % of GDP | CHE per capita in USD | Population per KM^2^ | Human Development Index (HDI) | HIV prevalence | DAHS per total HIV spending ratio | OOPE as % of the total HIV spending |
| --- | --- | --- | --- | --- | --- | --- | --- | --- | --- | --- |
| Rule of Law | 1.00 | 0.39 | 0.57 | 0.19 | 0.60 | 0.04 | 0.55 | -0.13 | -0.04 | -0.34 |
| Antenatal Care Coverage | 0.39 | 1.00 | 0.53 | 0.36 | 0.49 | 0.03 | 0.70 | -0.22 | 0.12 | -0.18 |
| GNI per capita in USD | 0.57 | 0.53 | 1.00 | 0.04 | 0.87 | -0.15 | 0.69 | -0.18 | -0.10 | -0.25 |
| CHE as % of GDP | 0.19 | 0.36 | 0.04 | 1.00 | 0.32 | 0.20 | 0.17 | 0.00 | -0.03 | -0.09 |
| CHE per capita in USD | 0.60 | 0.49 | 0.87 | 0.32 | 1.00 | -0.09 | 0.64 | -0.21 | -0.10 | -0.26 |
| Population per KM^2^ | 0.04 | 0.03 | -0.15 | 0.20 | -0.09 | 1.00 | -0.08 | -0.07 | -0.07 | -0.05 |
| Human Development Index (HDI) | 0.55 | 0.70 | 0.69 | 0.17 | 0.64 | -0.08 | 1.00 | -0.33 | -0.01 | -0.23 |
| HIV prevalence | -0.13 | -0.22 | -0.18 | 0.00 | -0.21 | -0.07 | -0.33 | 1.00 | -0.04 | -0.26 |
| DAHS per total HIV spending ratio | -0.04 | 0.12 | -0.10 | -0.03 | -0.10 | -0.07 | -0.01 | -0.04 | 1.00 | 0.06 |
| OOPE as % of the total HIV spending | -0.34 | -0.18 | -0.25 | -0.09 | -0.26 | -0.05 | -0.23 | -0.26 | 0.06 | 1.00 |

*Notes*: Red colours indicate negative correlation, whereas green colours a positive correlation. Yellow colours stand for weak but positive correlation. CHE: Catastrophic Health Expenditure, HDI: Human Development Index, GNI: Gross National Income, OOPE: Out-of-pocket expenditure, USD: United States Dollars.

**Table C.** Countries and number of observations per country (N=78 countries, n=657 observations)

| **N** | **Country code** | **Year** | **DEA** | **BIAS** | **DEA robust (adjusted)** | **Word Bank category** | **WHO group** |  |
| --- | --- | --- | --- | --- | --- | --- | --- | --- |
|  |  |  |  |  |  |  |  |  |
|  |  |  |  |  |  |  |  |  |
| 1 | DZA | 2018 | 1.000 | 0.056 | 0.944 | Upper middle income | AFRO |  |
| 2 | AGO | 2010 | 0.190 | 0.002 | 0.188 | Lower middle income | AFRO |  |
| 3 | AGO | 2011 | 0.151 | 0.004 | 0.147 | Lower middle income | AFRO |  |
| 4 | AGO | 2012 | 0.164 | 0.005 | 0.159 | Lower middle income | AFRO |  |
| 5 | AGO | 2013 | 0.265 | 0.004 | 0.260 | Lower middle income | AFRO |  |
| 6 | AGO | 2014 | 0.390 | 0.005 | 0.385 | Lower middle income | AFRO |  |
| 7 | AGO | 2015 | 0.365 | 0.006 | 0.359 | Lower middle income | AFRO |  |
| 8 | AGO | 2016 | 0.326 | 0.005 | 0.320 | Lower middle income | AFRO |  |
| 9 | AGO | 2017 | 0.315 | 0.009 | 0.307 | Lower middle income | AFRO |  |
| 10 | AGO | 2018 | 0.413 | 0.007 | 0.406 | Lower middle income | AFRO |  |
| 11 | ARG | 2010 | 0.950 | 0.002 | 0.948 | Upper middle income | PAHO |  |
| 12 | ARG | 2011 | 0.950 | 0.003 | 0.947 | Upper middle income | PAHO |  |
| 13 | ARG | 2012 | 0.950 | 0.004 | 0.946 | Upper middle income | PAHO |  |
| 14 | ARG | 2013 | 0.950 | 0.003 | 0.947 | Upper middle income | PAHO |  |
| 15 | ARG | 2014 | 0.950 | 0.004 | 0.946 | Upper middle income | PAHO |  |
| 16 | ARG | 2015 | 0.950 | 0.004 | 0.946 | Upper middle income | PAHO |  |
| 17 | ARG | 2016 | 0.950 | 0.005 | 0.945 | Upper middle income | PAHO |  |
| 18 | ARG | 2017 | 0.960 | 0.004 | 0.956 | Upper middle income | PAHO |  |
| 19 | ARG | 2018 | 0.940 | 0.006 | 0.934 | Upper middle income | PAHO |  |
| 20 | ARM | 2010 | 0.285 | 0.007 | 0.278 | Upper middle income | EURO |  |
| 21 | ARM | 2011 | 0.260 | 0.002 | 0.258 | Upper middle income | EURO |  |
| 22 | ARM | 2012 | 0.260 | 0.002 | 0.258 | Upper middle income | EURO |  |
| 23 | ARM | 2013 | 0.400 | 0.004 | 0.396 | Upper middle income | EURO |  |
| 24 | ARM | 2014 | 0.610 | 0.005 | 0.605 | Upper middle income | EURO |  |
| 25 | ARM | 2015 | 0.641 | 0.014 | 0.627 | Upper middle income | EURO |  |
| 26 | ARM | 2016 | 0.770 | 0.008 | 0.762 | Upper middle income | EURO |  |
| 27 | ARM | 2017 | 0.750 | 0.018 | 0.732 | Upper middle income | EURO |  |
| 28 | ARM | 2018 | 0.990 | 0.017 | 0.973 | Upper middle income | EURO |  |
| 29 | AZE | 2018 | 0.869 | 0.020 | 0.849 | Upper middle income | EURO |  |
| 30 | BLR | 2010 | 0.640 | 0.005 | 0.635 | Upper middle income | EURO |  |
| 31 | BLR | 2011 | 0.530 | 0.005 | 0.525 | Upper middle income | EURO |  |
| 32 | BLR | 2012 | 0.630 | 0.006 | 0.624 | Upper middle income | EURO |  |
| 33 | BLR | 2013 | 0.920 | 0.006 | 0.914 | Upper middle income | EURO |  |
| 34 | BLR | 2014 | 0.880 | 0.006 | 0.874 | Upper middle income | EURO |  |
| 35 | BLR | 2015 | 0.900 | 0.007 | 0.893 | Upper middle income | EURO |  |
| 36 | BLR | 2016 | 0.970 | 0.008 | 0.962 | Upper middle income | EURO |  |
| 37 | BLR | 2017 | 0.990 | 0.005 | 0.985 | Upper middle income | EURO |  |
| 38 | BLR | 2018 | 1.000 | 0.011 | 0.989 | Upper middle income | EURO |  |
| 39 | BEN | 2010 | 0.404 | 0.006 | 0.398 | Low income | AFRO |  |
| 40 | BEN | 2011 | 0.499 | 0.009 | 0.490 | Low income | AFRO |  |
| 41 | BEN | 2012 | 0.554 | 0.027 | 0.528 | Low income | AFRO |  |
| 42 | BEN | 2013 | 0.461 | 0.012 | 0.449 | Low income | AFRO |  |
| 43 | BEN | 2014 | 0.559 | 0.020 | 0.539 | Low income | AFRO |  |
| 44 | BEN | 2015 | 0.890 | 0.016 | 0.874 | Low income | AFRO |  |
| 45 | BEN | 2016 | 1.000 | 0.017 | 0.983 | Low income | AFRO |  |
| 46 | BEN | 2017 | 1.000 | 0.017 | 0.983 | Low income | AFRO |  |
| 47 | BEN | 2018 | 1.000 | 0.027 | 0.973 | Low income | AFRO |  |
| 48 | BOL | 2010 | 0.414 | 0.010 | 0.404 | Lower middle income | PAHO |  |
| 49 | BOL | 2011 | 0.535 | 0.012 | 0.523 | Lower middle income | PAHO |  |
| 50 | BOL | 2012 | 0.798 | 0.019 | 0.779 | Lower middle income | PAHO |  |
| 51 | BOL | 2013 | 0.660 | 0.004 | 0.656 | Lower middle income | PAHO |  |
| 52 | BOL | 2014 | 0.702 | 0.016 | 0.686 | Lower middle income | PAHO |  |
| 53 | BOL | 2015 | 0.846 | 0.022 | 0.825 | Lower middle income | PAHO |  |
| 54 | BOL | 2016 | 1.000 | 0.027 | 0.973 | Lower middle income | PAHO |  |
| 55 | BOL | 2017 | 0.956 | 0.023 | 0.933 | Lower middle income | PAHO |  |
| 56 | BOL | 2018 | 0.998 | 0.021 | 0.977 | Lower middle income | PAHO |  |
| 57 | BWA | 2010 | 0.750 | 0.002 | 0.748 | Upper middle income | AFRO |  |
| 58 | BWA | 2011 | 0.690 | 0.004 | 0.686 | Upper middle income | AFRO |  |
| 59 | BWA | 2012 | 0.860 | 0.003 | 0.857 | Upper middle income | AFRO |  |
| 60 | BWA | 2013 | 0.870 | 0.004 | 0.866 | Upper middle income | AFRO |  |
| 61 | BWA | 2014 | 0.800 | 0.009 | 0.791 | Upper middle income | AFRO |  |
| 62 | BWA | 2015 | 0.830 | 0.018 | 0.812 | Upper middle income | AFRO |  |
| 63 | BWA | 2016 | 0.830 | 0.038 | 0.792 | Upper middle income | AFRO |  |
| 64 | BWA | 2017 | 0.960 | 0.010 | 0.950 | Upper middle income | AFRO |  |
| 65 | BWA | 2018 | 1.000 | 0.015 | 0.985 | Upper middle income | AFRO |  |
| 66 | BFA | 2010 | 0.382 | 0.008 | 0.374 | Low income | AFRO |  |
| 67 | BFA | 2011 | 0.435 | 0.016 | 0.419 | Low income | AFRO |  |
| 68 | BFA | 2012 | 0.466 | 0.017 | 0.449 | Low income | AFRO |  |
| 69 | BFA | 2013 | 0.497 | 0.012 | 0.485 | Low income | AFRO |  |
| 70 | BFA | 2014 | 0.727 | 0.026 | 0.701 | Low income | AFRO |  |
| 71 | BFA | 2015 | 0.726 | 0.019 | 0.707 | Low income | AFRO |  |
| 72 | BFA | 2016 | 0.770 | 0.021 | 0.749 | Low income | AFRO |  |
| 73 | BFA | 2017 | 0.873 | 0.024 | 0.849 | Low income | AFRO |  |
| 74 | BFA | 2018 | 0.817 | 0.029 | 0.788 | Low income | AFRO |  |
| 75 | BDI | 2010 | 0.440 | 0.005 | 0.435 | Low income | AFRO |  |
| 76 | BDI | 2011 | 0.461 | 0.008 | 0.453 | Low income | AFRO |  |
| 77 | BDI | 2012 | 0.485 | 0.008 | 0.477 | Low income | AFRO |  |
| 78 | BDI | 2013 | 0.556 | 0.009 | 0.547 | Low income | AFRO |  |
| 79 | BDI | 2014 | 0.639 | 0.017 | 0.621 | Low income | AFRO |  |
| 80 | BDI | 2015 | 0.732 | 0.020 | 0.711 | Low income | AFRO |  |
| 81 | BDI | 2016 | 0.861 | 0.026 | 0.835 | Low income | AFRO |  |
| 82 | BDI | 2017 | 0.874 | 0.023 | 0.851 | Low income | AFRO |  |
| 83 | BDI | 2018 | 0.963 | 0.018 | 0.945 | Low income | AFRO |  |
| 84 | KHM | 2010 | 0.663 | 0.011 | 0.651 | Lower middle income | WPRO |  |
| 85 | KHM | 2011 | 0.706 | 0.011 | 0.695 | Lower middle income | WPRO |  |
| 86 | KHM | 2012 | 0.772 | 0.020 | 0.752 | Lower middle income | WPRO |  |
| 87 | KHM | 2013 | 0.792 | 0.016 | 0.776 | Lower middle income | WPRO |  |
| 88 | KHM | 2014 | 0.837 | 0.019 | 0.818 | Lower middle income | WPRO |  |
| 89 | KHM | 2015 | 0.885 | 0.038 | 0.847 | Lower middle income | WPRO |  |
| 90 | KHM | 2016 | 0.929 | 0.039 | 0.890 | Lower middle income | WPRO |  |
| 91 | KHM | 2017 | 1.000 | 0.043 | 0.957 | Lower middle income | WPRO |  |
| 92 | KHM | 2018 | 1.000 | 0.046 | 0.954 | Lower middle income | WPRO |  |
| 93 | CMR | 2010 | 0.440 | 0.004 | 0.436 | Lower middle income | AFRO |  |
| 94 | CMR | 2011 | 0.510 | 0.004 | 0.506 | Lower middle income | AFRO |  |
| 95 | CMR | 2012 | 0.600 | 0.003 | 0.597 | Lower middle income | AFRO |  |
| 96 | CMR | 2013 | 0.810 | 0.003 | 0.807 | Lower middle income | AFRO |  |
| 97 | CMR | 2014 | 0.810 | 0.003 | 0.807 | Lower middle income | AFRO |  |
| 98 | CMR | 2015 | 1.000 | 0.004 | 0.996 | Lower middle income | AFRO |  |
| 99 | CMR | 2016 | 0.910 | 0.004 | 0.906 | Lower middle income | AFRO |  |
| 100 | CMR | 2017 | 0.940 | 0.004 | 0.936 | Lower middle income | AFRO |  |
| 101 | CMR | 2018 | 0.910 | 0.006 | 0.904 | Lower middle income | AFRO |  |
| 102 | CAF | 2010 | 0.320 | 0.002 | 0.318 | Low income | AFRO |  |
| 103 | CAF | 2011 | 0.420 | 0.003 | 0.417 | Low income | AFRO |  |
| 104 | CAF | 2012 | 0.489 | 0.011 | 0.477 | Low income | AFRO |  |
| 105 | CAF | 2013 | 0.290 | 0.004 | 0.286 | Low income | AFRO |  |
| 106 | CAF | 2014 | 0.535 | 0.012 | 0.523 | Low income | AFRO |  |
| 107 | CAF | 2015 | 0.601 | 0.013 | 0.587 | Low income | AFRO |  |
| 108 | CAF | 2016 | 0.850 | 0.006 | 0.844 | Low income | AFRO |  |
| 109 | CAF | 2017 | 0.650 | 0.010 | 0.640 | Low income | AFRO |  |
| 110 | CAF | 2018 | 0.720 | 0.010 | 0.710 | Low income | AFRO |  |
| 111 | TCD | 2010 | 0.403 | 0.010 | 0.393 | Low income | AFRO |  |
| 112 | TCD | 2011 | 0.410 | 0.010 | 0.400 | Low income | AFRO |  |
| 113 | TCD | 2012 | 0.435 | 0.020 | 0.415 | Low income | AFRO |  |
| 114 | TCD | 2013 | 0.512 | 0.024 | 0.488 | Low income | AFRO |  |
| 115 | TCD | 2014 | 0.697 | 0.022 | 0.675 | Low income | AFRO |  |
| 116 | TCD | 2015 | 0.697 | 0.026 | 0.671 | Low income | AFRO |  |
| 117 | TCD | 2016 | 0.531 | 0.024 | 0.507 | Low income | AFRO |  |
| 118 | TCD | 2017 | 0.613 | 0.026 | 0.587 | Low income | AFRO |  |
| 119 | TCD | 2018 | 0.726 | 0.032 | 0.695 | Low income | AFRO |  |
| 120 | CHL | 2010 | 0.820 | 0.002 | 0.818 | High income | PAHO |  |
| 121 | CHL | 2011 | 0.740 | 0.002 | 0.738 | High income | PAHO |  |
| 122 | CHL | 2012 | 0.720 | 0.003 | 0.717 | High income | PAHO |  |
| 123 | CHL | 2013 | 1.000 | 0.003 | 0.997 | High income | PAHO |  |
| 124 | CHL | 2014 | 0.900 | 0.002 | 0.898 | High income | PAHO |  |
| 125 | CHL | 2015 | 0.790 | 0.004 | 0.786 | High income | PAHO |  |
| 126 | CHL | 2016 | 0.950 | 0.004 | 0.946 | High income | PAHO |  |
| 127 | CHL | 2017 | 0.890 | 0.007 | 0.883 | High income | PAHO |  |
| 128 | CHL | 2018 | 1.000 | 0.005 | 0.995 | High income | PAHO |  |
| 129 | COL | 2010 | 0.730 | 0.005 | 0.725 | Upper middle income | PAHO |  |
| 130 | COL | 2011 | 0.670 | 0.005 | 0.665 | Upper middle income | PAHO |  |
| 131 | COL | 2012 | 0.680 | 0.003 | 0.677 | Upper middle income | PAHO |  |
| 132 | COL | 2013 | 0.690 | 0.005 | 0.685 | Upper middle income | PAHO |  |
| 133 | COL | 2014 | 0.790 | 0.005 | 0.785 | Upper middle income | PAHO |  |
| 134 | COL | 2015 | 0.707 | 0.011 | 0.696 | Upper middle income | PAHO |  |
| 135 | COL | 2016 | 0.686 | 0.013 | 0.674 | Upper middle income | PAHO |  |
| 136 | COL | 2017 | 0.850 | 0.013 | 0.837 | Upper middle income | PAHO |  |
| 137 | COL | 2018 | 0.865 | 0.028 | 0.838 | Upper middle income | PAHO |  |
| 138 | CRI | 2010 | 0.436 | 0.012 | 0.424 | Upper middle income | PAHO |  |
| 139 | CRI | 2011 | 0.440 | 0.013 | 0.427 | Upper middle income | PAHO |  |
| 140 | CRI | 2012 | 0.430 | 0.011 | 0.419 | Upper middle income | PAHO |  |
| 141 | CRI | 2013 | 0.463 | 0.012 | 0.451 | Upper middle income | PAHO |  |
| 142 | CRI | 2014 | 0.492 | 0.010 | 0.483 | Upper middle income | PAHO |  |
| 143 | CRI | 2015 | 0.509 | 0.012 | 0.498 | Upper middle income | PAHO |  |
| 144 | CRI | 2016 | 0.552 | 0.014 | 0.539 | Upper middle income | PAHO |  |
| 145 | CRI | 2017 | 0.561 | 0.017 | 0.545 | Upper middle income | PAHO |  |
| 146 | CRI | 2018 | 0.596 | 0.014 | 0.582 | Upper middle income | PAHO |  |
| 147 | CUB | 2010 | 0.640 | 0.003 | 0.637 | Upper middle income | PAHO |  |
| 148 | CUB | 2011 | 1.000 | 0.003 | 0.997 | Upper middle income | PAHO |  |
| 149 | CUB | 2012 | 1.000 | 0.003 | 0.997 | Upper middle income | PAHO |  |
| 150 | CUB | 2013 | 0.740 | 0.004 | 0.736 | Upper middle income | PAHO |  |
| 151 | CUB | 2014 | 0.644 | 0.011 | 0.633 | Upper middle income | PAHO |  |
| 152 | CUB | 2015 | 0.970 | 0.004 | 0.966 | Upper middle income | PAHO |  |
| 153 | CUB | 2016 | 0.850 | 0.012 | 0.838 | Upper middle income | PAHO |  |
| 154 | CUB | 2017 | 0.890 | 0.012 | 0.878 | Upper middle income | PAHO |  |
| 155 | CUB | 2018 | 1.000 | 0.010 | 0.990 | Upper middle income | PAHO |  |
| 156 | CIV | 2010 | 0.460 | 0.001 | 0.459 | Lower middle income | AFRO |  |
| 157 | CIV | 2011 | 0.430 | 0.002 | 0.428 | Lower middle income | AFRO |  |
| 158 | CIV | 2012 | 0.570 | 0.002 | 0.568 | Lower middle income | AFRO |  |
| 159 | CIV | 2013 | 0.710 | 0.002 | 0.708 | Lower middle income | AFRO |  |
| 160 | CIV | 2014 | 0.820 | 0.002 | 0.818 | Lower middle income | AFRO |  |
| 161 | CIV | 2015 | 0.840 | 0.004 | 0.836 | Lower middle income | AFRO |  |
| 162 | CIV | 2016 | 0.830 | 0.003 | 0.827 | Lower middle income | AFRO |  |
| 163 | CIV | 2017 | 0.870 | 0.005 | 0.865 | Lower middle income | AFRO |  |
| 164 | CIV | 2018 | 0.860 | 0.008 | 0.852 | Lower middle income | AFRO |  |
| 165 | COD | 2010 | 0.123 | 0.003 | 0.120 | Low income | AFRO |  |
| 166 | COD | 2011 | 0.150 | 0.004 | 0.146 | Low income | AFRO |  |
| 167 | COD | 2012 | 0.185 | 0.003 | 0.182 | Low income | AFRO |  |
| 168 | COD | 2013 | 0.229 | 0.010 | 0.219 | Low income | AFRO |  |
| 169 | COD | 2014 | 0.363 | 0.010 | 0.353 | Low income | AFRO |  |
| 170 | COD | 2015 | 0.433 | 0.011 | 0.422 | Low income | AFRO |  |
| 171 | COD | 2016 | 0.483 | 0.015 | 0.468 | Low income | AFRO |  |
| 172 | COD | 2017 | 0.588 | 0.018 | 0.570 | Low income | AFRO |  |
| 173 | COD | 2018 | 0.703 | 0.017 | 0.686 | Low income | AFRO |  |
| 174 | DJI | 2010 | 0.134 | 0.004 | 0.131 | Lower middle income | EMRO |  |
| 175 | DJI | 2011 | 0.163 | 0.002 | 0.161 | Lower middle income | EMRO |  |
| 176 | DJI | 2012 | 0.183 | 0.004 | 0.178 | Lower middle income | EMRO |  |
| 177 | DJI | 2013 | 0.195 | 0.003 | 0.192 | Lower middle income | EMRO |  |
| 178 | DJI | 2014 | 0.310 | 0.005 | 0.305 | Lower middle income | EMRO |  |
| 179 | DJI | 2015 | 0.420 | 0.002 | 0.418 | Lower middle income | EMRO |  |
| 180 | DJI | 2016 | 0.520 | 0.003 | 0.517 | Lower middle income | EMRO |  |
| 181 | DJI | 2017 | 0.364 | 0.010 | 0.354 | Lower middle income | EMRO |  |
| 182 | DJI | 2018 | 0.464 | 0.008 | 0.456 | Lower middle income | EMRO |  |
| 183 | DOM | 2010 | 0.540 | 0.001 | 0.539 | Upper middle income | PAHO |  |
| 184 | DOM | 2011 | 0.810 | 0.002 | 0.808 | Upper middle income | PAHO |  |
| 185 | DOM | 2012 | 1.000 | 0.002 | 0.998 | Upper middle income | PAHO |  |
| 186 | DOM | 2013 | 0.830 | 0.002 | 0.828 | Upper middle income | PAHO |  |
| 187 | DOM | 2014 | 0.980 | 0.003 | 0.977 | Upper middle income | PAHO |  |
| 188 | DOM | 2015 | 0.870 | 0.003 | 0.867 | Upper middle income | PAHO |  |
| 189 | DOM | 2016 | 0.940 | 0.003 | 0.937 | Upper middle income | PAHO |  |
| 190 | DOM | 2017 | 0.890 | 0.004 | 0.886 | Upper middle income | PAHO |  |
| 191 | DOM | 2018 | 0.890 | 0.007 | 0.883 | Upper middle income | PAHO |  |
| 192 | ECU | 2010 | 0.519 | 0.011 | 0.508 | Upper middle income | PAHO |  |
| 193 | ECU | 2011 | 0.561 | 0.012 | 0.549 | Upper middle income | PAHO |  |
| 194 | ECU | 2012 | 0.588 | 0.012 | 0.575 | Upper middle income | PAHO |  |
| 195 | ECU | 2013 | 0.593 | 0.013 | 0.580 | Upper middle income | PAHO |  |
| 196 | ECU | 2014 | 0.618 | 0.015 | 0.603 | Upper middle income | PAHO |  |
| 197 | ECU | 2015 | 0.638 | 0.019 | 0.619 | Upper middle income | PAHO |  |
| 198 | ECU | 2016 | 0.656 | 0.030 | 0.626 | Upper middle income | PAHO |  |
| 199 | ECU | 2017 | 0.718 | 0.028 | 0.689 | Upper middle income | PAHO |  |
| 200 | ECU | 2018 | 0.782 | 0.028 | 0.753 | Upper middle income | PAHO |  |
| 201 | SLV | 2010 | 0.384 | 0.009 | 0.374 | Lower middle income | PAHO |  |
| 202 | SLV | 2011 | 0.402 | 0.012 | 0.390 | Lower middle income | PAHO |  |
| 203 | SLV | 2012 | 0.500 | 0.008 | 0.493 | Lower middle income | PAHO |  |
| 204 | SLV | 2013 | 0.590 | 0.004 | 0.586 | Lower middle income | PAHO |  |
| 205 | SLV | 2014 | 0.580 | 0.008 | 0.572 | Lower middle income | PAHO |  |
| 206 | SLV | 2015 | 0.562 | 0.008 | 0.554 | Lower middle income | PAHO |  |
| 207 | SLV | 2016 | 0.541 | 0.014 | 0.527 | Lower middle income | PAHO |  |
| 208 | SLV | 2017 | 0.555 | 0.015 | 0.540 | Lower middle income | PAHO |  |
| 209 | SLV | 2018 | 0.548 | 0.017 | 0.531 | Lower middle income | PAHO |  |
| 210 | GNQ | 2010 | 0.160 | 0.001 | 0.159 | Upper middle income | AFRO |  |
| 211 | GNQ | 2011 | 0.134 | 0.002 | 0.132 | Upper middle income | AFRO |  |
| 212 | GNQ | 2012 | 0.177 | 0.003 | 0.174 | Upper middle income | AFRO |  |
| 213 | GNQ | 2013 | 0.590 | 0.002 | 0.588 | Upper middle income | AFRO |  |
| 214 | GNQ | 2014 | 0.560 | 0.002 | 0.558 | Upper middle income | AFRO |  |
| 215 | GNQ | 2015 | 0.440 | 0.001 | 0.439 | Upper middle income | AFRO |  |
| 216 | GNQ | 2016 | 0.670 | 0.002 | 0.668 | Upper middle income | AFRO |  |
| 217 | GNQ | 2017 | 0.610 | 0.003 | 0.607 | Upper middle income | AFRO |  |
| 218 | GNQ | 2018 | 0.491 | 0.007 | 0.484 | Upper middle income | AFRO |  |
| 219 | ERI | 2010 | 0.341 | 0.005 | 0.336 | Low income | AFRO |  |
| 220 | ERI | 2011 | 0.408 | 0.011 | 0.397 | Low income | AFRO |  |
| 221 | ERI | 2012 | 0.494 | 0.009 | 0.485 | Low income | AFRO |  |
| 222 | ERI | 2013 | 0.549 | 0.020 | 0.529 | Low income | AFRO |  |
| 223 | ERI | 2014 | 0.609 | 0.010 | 0.600 | Low income | AFRO |  |
| 224 | ERI | 2015 | 0.653 | 0.025 | 0.628 | Low income | AFRO |  |
| 225 | ERI | 2016 | 0.596 | 0.015 | 0.581 | Low income | AFRO |  |
| 226 | ERI | 2017 | 0.599 | 0.011 | 0.588 | Low income | AFRO |  |
| 227 | ERI | 2018 | 0.606 | 0.012 | 0.594 | Low income | AFRO |  |
| 228 | SWZ | 2010 | 0.660 | 0.001 | 0.659 | Lower middle income | AFRO |  |
| 229 | SWZ | 2011 | 0.840 | 0.001 | 0.839 | Lower middle income | AFRO |  |
| 230 | SWZ | 2012 | 0.840 | 0.002 | 0.838 | Lower middle income | AFRO |  |
| 231 | SWZ | 2013 | 0.860 | 0.002 | 0.858 | Lower middle income | AFRO |  |
| 232 | SWZ | 2014 | 0.800 | 0.004 | 0.796 | Lower middle income | AFRO |  |
| 233 | SWZ | 2015 | 0.870 | 0.009 | 0.861 | Lower middle income | AFRO |  |
| 234 | SWZ | 2016 | 0.910 | 0.018 | 0.892 | Lower middle income | AFRO |  |
| 235 | SWZ | 2017 | 0.948 | 0.034 | 0.914 | Lower middle income | AFRO |  |
| 236 | SWZ | 2018 | 0.970 | 0.024 | 0.946 | Lower middle income | AFRO |  |
| 237 | ETH | 2010 | 0.425 | 0.007 | 0.418 | Low income | AFRO |  |
| 238 | ETH | 2011 | 0.501 | 0.009 | 0.492 | Low income | AFRO |  |
| 239 | ETH | 2012 | 0.622 | 0.011 | 0.611 | Low income | AFRO |  |
| 240 | ETH | 2013 | 0.723 | 0.011 | 0.712 | Low income | AFRO |  |
| 241 | ETH | 2014 | 0.756 | 0.013 | 0.742 | Low income | AFRO |  |
| 242 | ETH | 2015 | 0.822 | 0.013 | 0.809 | Low income | AFRO |  |
| 243 | ETH | 2016 | 0.778 | 0.023 | 0.755 | Low income | AFRO |  |
| 244 | ETH | 2017 | 0.792 | 0.032 | 0.760 | Low income | AFRO |  |
| 245 | ETH | 2018 | 0.894 | 0.028 | 0.866 | Low income | AFRO |  |
| 246 | GAB | 2010 | 0.311 | 0.005 | 0.306 | Upper middle income | AFRO |  |
| 247 | GAB | 2011 | 0.342 | 0.007 | 0.334 | Upper middle income | AFRO |  |
| 248 | GAB | 2012 | 0.395 | 0.011 | 0.384 | Upper middle income | AFRO |  |
| 249 | GAB | 2013 | 0.500 | 0.013 | 0.487 | Upper middle income | AFRO |  |
| 250 | GAB | 2014 | 0.531 | 0.012 | 0.519 | Upper middle income | AFRO |  |
| 251 | GAB | 2015 | 0.638 | 0.014 | 0.623 | Upper middle income | AFRO |  |
| 252 | GAB | 2016 | 0.714 | 0.017 | 0.698 | Upper middle income | AFRO |  |
| 253 | GAB | 2017 | 0.746 | 0.015 | 0.731 | Upper middle income | AFRO |  |
| 254 | GAB | 2018 | 0.799 | 0.017 | 0.782 | Upper middle income | AFRO |  |
| 255 | GMB | 2010 | 0.590 | 0.002 | 0.588 | Low income | AFRO |  |
| 256 | GMB | 2011 | 0.790 | 0.002 | 0.788 | Low income | AFRO |  |
| 257 | GMB | 2012 | 0.990 | 0.003 | 0.987 | Low income | AFRO |  |
| 258 | GMB | 2013 | 0.650 | 0.002 | 0.648 | Low income | AFRO |  |
| 259 | GMB | 2014 | 0.630 | 0.002 | 0.628 | Low income | AFRO |  |
| 260 | GMB | 2015 | 0.740 | 0.005 | 0.735 | Low income | AFRO |  |
| 261 | GMB | 2016 | 0.630 | 0.006 | 0.624 | Low income | AFRO |  |
| 262 | GMB | 2017 | 0.600 | 0.003 | 0.597 | Low income | AFRO |  |
| 263 | GMB | 2018 | 0.560 | 0.004 | 0.556 | Low income | AFRO |  |
| 264 | GEO | 2010 | 0.530 | 0.002 | 0.528 | Upper middle income | EURO |  |
| 265 | GEO | 2011 | 0.410 | 0.002 | 0.408 | Upper middle income | EURO |  |
| 266 | GEO | 2012 | 0.329 | 0.006 | 0.323 | Upper middle income | EURO |  |
| 267 | GEO | 2013 | 0.510 | 0.003 | 0.507 | Upper middle income | EURO |  |
| 268 | GEO | 2014 | 0.630 | 0.004 | 0.626 | Upper middle income | EURO |  |
| 269 | GEO | 2015 | 0.920 | 0.003 | 0.917 | Upper middle income | EURO |  |
| 270 | GEO | 2016 | 0.660 | 0.008 | 0.652 | Upper middle income | EURO |  |
| 271 | GEO | 2017 | 0.800 | 0.005 | 0.795 | Upper middle income | EURO |  |
| 272 | GEO | 2018 | 0.584 | 0.010 | 0.574 | Upper middle income | EURO |  |
| 273 | GHA | 2010 | 0.370 | 0.001 | 0.369 | Lower middle income | AFRO |  |
| 274 | GHA | 2011 | 0.500 | 0.001 | 0.499 | Lower middle income | AFRO |  |
| 275 | GHA | 2012 | 0.550 | 0.002 | 0.548 | Lower middle income | AFRO |  |
| 276 | GHA | 2013 | 0.440 | 0.002 | 0.438 | Lower middle income | AFRO |  |
| 277 | GHA | 2014 | 0.510 | 0.003 | 0.507 | Lower middle income | AFRO |  |
| 278 | GHA | 2015 | 0.480 | 0.003 | 0.477 | Lower middle income | AFRO |  |
| 279 | GHA | 2016 | 0.610 | 0.003 | 0.607 | Lower middle income | AFRO |  |
| 280 | GHA | 2017 | 0.670 | 0.003 | 0.667 | Lower middle income | AFRO |  |
| 281 | GHA | 2018 | 0.730 | 0.003 | 0.727 | Lower middle income | AFRO |  |
| 282 | GTM | 2010 | 0.346 | 0.005 | 0.340 | Upper middle income | PAHO |  |
| 283 | GTM | 2011 | 0.440 | 0.006 | 0.434 | Upper middle income | PAHO |  |
| 284 | GTM | 2012 | 0.380 | 0.009 | 0.371 | Upper middle income | PAHO |  |
| 285 | GTM | 2013 | 0.520 | 0.008 | 0.512 | Upper middle income | PAHO |  |
| 286 | GTM | 2014 | 0.490 | 0.009 | 0.482 | Upper middle income | PAHO |  |
| 287 | GTM | 2015 | 0.422 | 0.008 | 0.414 | Upper middle income | PAHO |  |
| 288 | GTM | 2016 | 0.456 | 0.012 | 0.444 | Upper middle income | PAHO |  |
| 289 | GTM | 2017 | 0.469 | 0.014 | 0.455 | Upper middle income | PAHO |  |
| 290 | GTM | 2018 | 0.550 | 0.014 | 0.536 | Upper middle income | PAHO |  |
| 291 | GNB | 2010 | 0.180 | 0.001 | 0.179 | Low income | AFRO |  |
| 292 | GNB | 2011 | 0.250 | 0.002 | 0.248 | Low income | AFRO |  |
| 293 | GNB | 2012 | 0.380 | 0.004 | 0.376 | Low income | AFRO |  |
| 294 | GNB | 2013 | 0.610 | 0.001 | 0.609 | Low income | AFRO |  |
| 295 | GNB | 2014 | 0.980 | 0.006 | 0.974 | Low income | AFRO |  |
| 296 | GNB | 2015 | 0.820 | 0.003 | 0.817 | Low income | AFRO |  |
| 297 | GNB | 2016 | 0.780 | 0.003 | 0.777 | Low income | AFRO |  |
| 298 | GNB | 2017 | 0.760 | 0.003 | 0.757 | Low income | AFRO |  |
| 299 | GNB | 2018 | 0.560 | 0.005 | 0.555 | Low income | AFRO |  |
| 300 | HTI | 2010 | 0.370 | 0.001 | 0.369 | Low income | PAHO |  |
| 301 | HTI | 2011 | 0.610 | 0.001 | 0.609 | Low income | PAHO |  |
| 302 | HTI | 2012 | 0.800 | 0.002 | 0.798 | Low income | PAHO |  |
| 303 | HTI | 2013 | 0.860 | 0.002 | 0.858 | Low income | PAHO |  |
| 304 | HTI | 2014 | 0.740 | 0.002 | 0.738 | Low income | PAHO |  |
| 305 | HTI | 2015 | 0.760 | 0.003 | 0.757 | Low income | PAHO |  |
| 306 | HTI | 2016 | 0.731 | 0.010 | 0.720 | Low income | PAHO |  |
| 307 | HTI | 2017 | 0.772 | 0.011 | 0.761 | Low income | PAHO |  |
| 308 | HTI | 2018 | 0.840 | 0.009 | 0.831 | Low income | PAHO |  |
| 309 | HND | 2010 | 0.710 | 0.003 | 0.707 | Lower middle income | PAHO |  |
| 310 | HND | 2011 | 0.710 | 0.003 | 0.707 | Lower middle income | PAHO |  |
| 311 | HND | 2012 | 0.770 | 0.003 | 0.767 | Lower middle income | PAHO |  |
| 312 | HND | 2013 | 0.700 | 0.006 | 0.694 | Lower middle income | PAHO |  |
| 313 | HND | 2014 | 0.670 | 0.011 | 0.659 | Lower middle income | PAHO |  |
| 314 | HND | 2015 | 0.620 | 0.010 | 0.610 | Lower middle income | PAHO |  |
| 315 | HND | 2016 | 0.611 | 0.014 | 0.597 | Lower middle income | PAHO |  |
| 316 | HND | 2017 | 0.659 | 0.013 | 0.646 | Lower middle income | PAHO |  |
| 317 | HND | 2018 | 0.739 | 0.012 | 0.727 | Lower middle income | PAHO |  |
| 318 | IDN | 2017 | 0.201 | 0.008 | 0.193 | Lower middle income | SEARO |  |
| 319 | IDN | 2018 | 0.236 | 0.009 | 0.227 | Lower middle income | SEARO |  |
| 320 | IRN | 2010 | 0.153 | 0.004 | 0.149 | Upper middle income | EMRO |  |
| 321 | IRN | 2011 | 0.185 | 0.005 | 0.180 | Upper middle income | EMRO |  |
| 322 | IRN | 2012 | 0.305 | 0.007 | 0.298 | Upper middle income | EMRO |  |
| 323 | IRN | 2013 | 0.326 | 0.008 | 0.318 | Upper middle income | EMRO |  |
| 324 | IRN | 2014 | 0.407 | 0.010 | 0.398 | Upper middle income | EMRO |  |
| 325 | IRN | 2015 | 0.539 | 0.012 | 0.527 | Upper middle income | EMRO |  |
| 326 | IRN | 2016 | 0.610 | 0.004 | 0.606 | Upper middle income | EMRO |  |
| 327 | IRN | 2017 | 0.670 | 0.005 | 0.665 | Upper middle income | EMRO |  |
| 328 | IRN | 2018 | 0.780 | 0.005 | 0.775 | Upper middle income | EMRO |  |
| 329 | KAZ | 2010 | 0.899 | 0.021 | 0.878 | Upper middle income | EURO |  |
| 330 | KAZ | 2011 | 0.990 | 0.023 | 0.967 | Upper middle income | EURO |  |
| 331 | KAZ | 2012 | 0.950 | 0.006 | 0.944 | Upper middle income | EURO |  |
| 332 | KAZ | 2013 | 0.974 | 0.022 | 0.952 | Upper middle income | EURO |  |
| 333 | KAZ | 2014 | 0.986 | 0.022 | 0.964 | Upper middle income | EURO |  |
| 334 | KAZ | 2015 | 0.920 | 0.007 | 0.913 | Upper middle income | EURO |  |
| 335 | KAZ | 2016 | 0.970 | 0.008 | 0.962 | Upper middle income | EURO |  |
| 336 | KAZ | 2017 | 0.980 | 0.012 | 0.968 | Upper middle income | EURO |  |
| 337 | KAZ | 2018 | 0.990 | 0.023 | 0.967 | Upper middle income | EURO |  |
| 338 | KEN | 2010 | 0.490 | 0.002 | 0.488 | Lower middle income | AFRO |  |
| 339 | KEN | 2011 | 0.680 | 0.002 | 0.678 | Lower middle income | AFRO |  |
| 340 | KEN | 2012 | 0.550 | 0.005 | 0.545 | Lower middle income | AFRO |  |
| 341 | KEN | 2013 | 0.570 | 0.008 | 0.562 | Lower middle income | AFRO |  |
| 342 | KEN | 2014 | 0.670 | 0.007 | 0.663 | Lower middle income | AFRO |  |
| 343 | KEN | 2015 | 0.850 | 0.006 | 0.844 | Lower middle income | AFRO |  |
| 344 | KEN | 2016 | 0.870 | 0.015 | 0.855 | Lower middle income | AFRO |  |
| 345 | KEN | 2017 | 0.848 | 0.033 | 0.814 | Lower middle income | AFRO |  |
| 346 | KEN | 2018 | 0.880 | 0.011 | 0.869 | Lower middle income | AFRO |  |
| 347 | KGZ | 2010 | 0.690 | 0.004 | 0.686 | Lower middle income | EURO |  |
| 348 | KGZ | 2011 | 0.480 | 0.002 | 0.478 | Lower middle income | EURO |  |
| 349 | KGZ | 2012 | 0.810 | 0.003 | 0.807 | Lower middle income | EURO |  |
| 350 | KGZ | 2013 | 0.830 | 0.003 | 0.827 | Lower middle income | EURO |  |
| 351 | KGZ | 2014 | 0.860 | 0.003 | 0.857 | Lower middle income | EURO |  |
| 352 | KGZ | 2015 | 0.610 | 0.003 | 0.607 | Lower middle income | EURO |  |
| 353 | KGZ | 2016 | 0.750 | 0.004 | 0.746 | Lower middle income | EURO |  |
| 354 | KGZ | 2017 | 0.770 | 0.005 | 0.765 | Lower middle income | EURO |  |
| 355 | KGZ | 2018 | 0.820 | 0.007 | 0.813 | Lower middle income | EURO |  |
| 356 | LAO | 2010 | 0.228 | 0.011 | 0.216 | Lower middle income | WPRO |  |
| 357 | LAO | 2011 | 0.261 | 0.011 | 0.249 | Lower middle income | WPRO |  |
| 358 | LAO | 2012 | 0.310 | 0.012 | 0.298 | Lower middle income | WPRO |  |
| 359 | LAO | 2013 | 0.319 | 0.012 | 0.308 | Lower middle income | WPRO |  |
| 360 | LAO | 2014 | 0.388 | 0.019 | 0.369 | Lower middle income | WPRO |  |
| 361 | LAO | 2015 | 0.449 | 0.022 | 0.427 | Lower middle income | WPRO |  |
| 362 | LAO | 2016 | 0.537 | 0.020 | 0.517 | Lower middle income | WPRO |  |
| 363 | LAO | 2017 | 0.625 | 0.024 | 0.601 | Lower middle income | WPRO |  |
| 364 | LAO | 2018 | 0.724 | 0.027 | 0.697 | Lower middle income | WPRO |  |
| 365 | LSO | 2010 | 0.640 | 0.001 | 0.639 | Lower middle income | AFRO |  |
| 366 | LSO | 2011 | 0.870 | 0.001 | 0.869 | Lower middle income | AFRO |  |
| 367 | LSO | 2012 | 0.860 | 0.001 | 0.859 | Lower middle income | AFRO |  |
| 368 | LSO | 2013 | 0.820 | 0.001 | 0.819 | Lower middle income | AFRO |  |
| 369 | LSO | 2014 | 0.880 | 0.001 | 0.879 | Lower middle income | AFRO |  |
| 370 | LSO | 2015 | 0.890 | 0.002 | 0.888 | Lower middle income | AFRO |  |
| 371 | LSO | 2016 | 0.830 | 0.002 | 0.828 | Lower middle income | AFRO |  |
| 372 | LSO | 2017 | 0.780 | 0.006 | 0.774 | Lower middle income | AFRO |  |
| 373 | LSO | 2018 | 0.860 | 0.004 | 0.856 | Lower middle income | AFRO |  |
| 374 | LBR | 2010 | 0.210 | 0.001 | 0.209 | Low income | AFRO |  |
| 375 | LBR | 2011 | 0.320 | 0.001 | 0.319 | Low income | AFRO |  |
| 376 | LBR | 2012 | 0.340 | 0.001 | 0.339 | Low income | AFRO |  |
| 377 | LBR | 2013 | 0.270 | 0.002 | 0.268 | Low income | AFRO |  |
| 378 | LBR | 2014 | 0.280 | 0.005 | 0.275 | Low income | AFRO |  |
| 379 | LBR | 2015 | 0.530 | 0.002 | 0.528 | Low income | AFRO |  |
| 380 | LBR | 2016 | 0.640 | 0.002 | 0.638 | Low income | AFRO |  |
| 381 | LBR | 2017 | 0.740 | 0.002 | 0.738 | Low income | AFRO |  |
| 382 | LBR | 2018 | 0.890 | 0.003 | 0.887 | Low income | AFRO |  |
| 383 | MWI | 2010 | 0.335 | 0.011 | 0.324 | Low income | AFRO |  |
| 384 | MWI | 2011 | 0.490 | 0.008 | 0.482 | Low income | AFRO |  |
| 385 | MWI | 2012 | 0.670 | 0.004 | 0.666 | Low income | AFRO |  |
| 386 | MWI | 2013 | 0.690 | 0.008 | 0.682 | Low income | AFRO |  |
| 387 | MWI | 2014 | 0.760 | 0.010 | 0.750 | Low income | AFRO |  |
| 388 | MWI | 2015 | 0.820 | 0.010 | 0.810 | Low income | AFRO |  |
| 389 | MWI | 2016 | 0.890 | 0.014 | 0.876 | Low income | AFRO |  |
| 390 | MWI | 2017 | 0.990 | 0.011 | 0.979 | Low income | AFRO |  |
| 391 | MWI | 2018 | 1.000 | 0.026 | 0.974 | Low income | AFRO |  |
| 392 | MYS | 2010 | 0.801 | 0.018 | 0.783 | Upper middle income | WPRO |  |
| 393 | MYS | 2011 | 0.842 | 0.019 | 0.823 | Upper middle income | WPRO |  |
| 394 | MYS | 2012 | 0.822 | 0.018 | 0.804 | Upper middle income | WPRO |  |
| 395 | MYS | 2013 | 0.784 | 0.019 | 0.765 | Upper middle income | WPRO |  |
| 396 | MYS | 2014 | 0.830 | 0.006 | 0.824 | Upper middle income | WPRO |  |
| 397 | MYS | 2015 | 0.960 | 0.007 | 0.953 | Upper middle income | WPRO |  |
| 398 | MYS | 2016 | 0.990 | 0.010 | 0.980 | Upper middle income | WPRO |  |
| 399 | MYS | 2017 | 0.970 | 0.011 | 0.959 | Upper middle income | WPRO |  |
| 400 | MYS | 2018 | 0.990 | 0.012 | 0.978 | Upper middle income | WPRO |  |
| 401 | MLI | 2010 | 0.288 | 0.009 | 0.279 | Low income | AFRO |  |
| 402 | MLI | 2011 | 0.393 | 0.010 | 0.382 | Low income | AFRO |  |
| 403 | MLI | 2012 | 0.410 | 0.004 | 0.406 | Low income | AFRO |  |
| 404 | MLI | 2013 | 0.315 | 0.012 | 0.303 | Low income | AFRO |  |
| 405 | MLI | 2014 | 0.305 | 0.011 | 0.294 | Low income | AFRO |  |
| 406 | MLI | 2015 | 0.364 | 0.016 | 0.348 | Low income | AFRO |  |
| 407 | MLI | 2016 | 0.340 | 0.011 | 0.330 | Low income | AFRO |  |
| 408 | MLI | 2017 | 0.352 | 0.010 | 0.341 | Low income | AFRO |  |
| 409 | MLI | 2018 | 0.392 | 0.010 | 0.382 | Low income | AFRO |  |
| 410 | MRT | 2010 | 0.288 | 0.007 | 0.282 | Lower middle income | AFRO |  |
| 411 | MRT | 2011 | 0.313 | 0.007 | 0.306 | Lower middle income | AFRO |  |
| 412 | MRT | 2012 | 0.338 | 0.008 | 0.330 | Lower middle income | AFRO |  |
| 413 | MRT | 2013 | 0.405 | 0.010 | 0.396 | Lower middle income | AFRO |  |
| 414 | MRT | 2014 | 0.890 | 0.008 | 0.882 | Lower middle income | AFRO |  |
| 415 | MRT | 2015 | 0.919 | 0.019 | 0.900 | Lower middle income | AFRO |  |
| 416 | MRT | 2016 | 0.549 | 0.013 | 0.536 | Lower middle income | AFRO |  |
| 417 | MRT | 2017 | 0.657 | 0.032 | 0.626 | Lower middle income | AFRO |  |
| 418 | MRT | 2018 | 0.683 | 0.036 | 0.647 | Lower middle income | AFRO |  |
| 419 | MEX | 2010 | 0.442 | 0.007 | 0.434 | Upper middle income | PAHO |  |
| 420 | MEX | 2011 | 0.462 | 0.008 | 0.454 | Upper middle income | PAHO |  |
| 421 | MEX | 2012 | 0.540 | 0.014 | 0.526 | Upper middle income | PAHO |  |
| 422 | MEX | 2013 | 0.780 | 0.005 | 0.775 | Upper middle income | PAHO |  |
| 423 | MEX | 2014 | 0.810 | 0.005 | 0.805 | Upper middle income | PAHO |  |
| 424 | MEX | 2015 | 0.950 | 0.006 | 0.944 | Upper middle income | PAHO |  |
| 425 | MEX | 2016 | 0.803 | 0.011 | 0.792 | Upper middle income | PAHO |  |
| 426 | MEX | 2017 | 0.857 | 0.012 | 0.845 | Upper middle income | PAHO |  |
| 427 | MEX | 2018 | 0.878 | 0.020 | 0.858 | Upper middle income | PAHO |  |
| 428 | MAR | 2014 | 0.665 | 0.019 | 0.646 | Lower middle income | EMRO |  |
| 429 | MOZ | 2010 | 0.240 | 0.001 | 0.239 | Low income | AFRO |  |
| 430 | MOZ | 2011 | 0.500 | 0.001 | 0.499 | Low income | AFRO |  |
| 431 | MOZ | 2012 | 0.780 | 0.002 | 0.778 | Low income | AFRO |  |
| 432 | MOZ | 2013 | 0.830 | 0.002 | 0.828 | Low income | AFRO |  |
| 433 | MOZ | 2014 | 0.920 | 0.002 | 0.918 | Low income | AFRO |  |
| 434 | MOZ | 2015 | 0.950 | 0.002 | 0.948 | Low income | AFRO |  |
| 435 | MOZ | 2016 | 0.890 | 0.002 | 0.888 | Low income | AFRO |  |
| 436 | MOZ | 2017 | 1.000 | 0.002 | 0.998 | Low income | AFRO |  |
| 437 | MOZ | 2018 | 1.000 | 0.003 | 0.997 | Low income | AFRO |  |
| 438 | MMR | 2010 | 0.367 | 0.008 | 0.359 | Lower middle income | SEARO |  |
| 439 | MMR | 2012 | 0.535 | 0.011 | 0.524 | Lower middle income | SEARO |  |
| 440 | MMR | 2013 | 0.591 | 0.014 | 0.577 | Lower middle income | SEARO |  |
| 441 | MMR | 2014 | 0.707 | 0.017 | 0.689 | Lower middle income | SEARO |  |
| 442 | MMR | 2016 | 0.883 | 0.029 | 0.854 | Lower middle income | SEARO |  |
| 443 | MMR | 2017 | 0.919 | 0.037 | 0.882 | Lower middle income | SEARO |  |
| 444 | MMR | 2018 | 1.000 | 0.050 | 0.950 | Lower middle income | SEARO |  |
| 445 | NAM | 2010 | 0.650 | 0.005 | 0.645 | Upper middle income | AFRO |  |
| 446 | NAM | 2011 | 0.710 | 0.009 | 0.701 | Upper middle income | AFRO |  |
| 447 | NAM | 2012 | 0.707 | 0.025 | 0.682 | Upper middle income | AFRO |  |
| 448 | NAM | 2013 | 0.780 | 0.016 | 0.764 | Upper middle income | AFRO |  |
| 449 | NAM | 2014 | 0.900 | 0.006 | 0.894 | Upper middle income | AFRO |  |
| 450 | NAM | 2015 | 0.980 | 0.007 | 0.973 | Upper middle income | AFRO |  |
| 451 | NAM | 2016 | 1.000 | 0.005 | 0.995 | Upper middle income | AFRO |  |
| 452 | NAM | 2017 | 1.000 | 0.015 | 0.985 | Upper middle income | AFRO |  |
| 453 | NAM | 2018 | 1.000 | 0.046 | 0.954 | Upper middle income | AFRO |  |
| 454 | NPL | 2010 | 0.217 | 0.008 | 0.208 | Low income | SEARO |  |
| 455 | NPL | 2011 | 0.303 | 0.012 | 0.290 | Low income | SEARO |  |
| 456 | NPL | 2012 | 0.374 | 0.017 | 0.358 | Low income | SEARO |  |
| 457 | NPL | 2013 | 0.450 | 0.016 | 0.434 | Low income | SEARO |  |
| 458 | NPL | 2014 | 0.548 | 0.017 | 0.531 | Low income | SEARO |  |
| 459 | NPL | 2015 | 0.557 | 0.027 | 0.530 | Low income | SEARO |  |
| 460 | NER | 2011 | 0.692 | 0.016 | 0.676 | Low income | AFRO |  |
| 461 | NER | 2012 | 0.633 | 0.014 | 0.618 | Low income | AFRO |  |
| 462 | NER | 2013 | 0.638 | 0.015 | 0.623 | Low income | AFRO |  |
| 463 | NER | 2014 | 0.742 | 0.016 | 0.726 | Low income | AFRO |  |
| 464 | NER | 2015 | 0.543 | 0.020 | 0.523 | Low income | AFRO |  |
| 465 | NER | 2016 | 0.843 | 0.020 | 0.823 | Low income | AFRO |  |
| 466 | NER | 2017 | 0.701 | 0.026 | 0.675 | Low income | AFRO |  |
| 467 | NER | 2018 | 0.736 | 0.027 | 0.709 | Low income | AFRO |  |
| 468 | NGA | 2010 | 0.289 | 0.009 | 0.281 | Lower middle income | AFRO |  |
| 469 | NGA | 2011 | 0.339 | 0.007 | 0.332 | Lower middle income | AFRO |  |
| 470 | NGA | 2012 | 0.415 | 0.010 | 0.405 | Lower middle income | AFRO |  |
| 471 | NGA | 2013 | 0.534 | 0.008 | 0.526 | Lower middle income | AFRO |  |
| 472 | NGA | 2014 | 0.625 | 0.010 | 0.615 | Lower middle income | AFRO |  |
| 473 | NGA | 2015 | 0.577 | 0.019 | 0.558 | Lower middle income | AFRO |  |
| 474 | NGA | 2016 | 0.628 | 0.026 | 0.602 | Lower middle income | AFRO |  |
| 475 | NGA | 2017 | 0.634 | 0.021 | 0.613 | Lower middle income | AFRO |  |
| 476 | NGA | 2018 | 0.660 | 0.017 | 0.643 | Lower middle income | AFRO |  |
| 477 | PNG | 2010 | 0.245 | 0.004 | 0.240 | Lower middle income | WPRO |  |
| 478 | PNG | 2011 | 0.294 | 0.007 | 0.287 | Lower middle income | WPRO |  |
| 479 | PNG | 2012 | 0.358 | 0.011 | 0.347 | Lower middle income | WPRO |  |
| 480 | PNG | 2013 | 0.436 | 0.014 | 0.422 | Lower middle income | WPRO |  |
| 481 | PNG | 2014 | 0.524 | 0.017 | 0.507 | Lower middle income | WPRO |  |
| 482 | PNG | 2015 | 0.607 | 0.013 | 0.594 | Lower middle income | WPRO |  |
| 483 | PNG | 2016 | 0.651 | 0.014 | 0.637 | Lower middle income | WPRO |  |
| 484 | PNG | 2017 | 0.720 | 0.012 | 0.708 | Lower middle income | WPRO |  |
| 485 | PNG | 2018 | 0.784 | 0.015 | 0.768 | Lower middle income | WPRO |  |
| 486 | PRY | 2010 | 0.500 | 0.003 | 0.497 | Upper middle income | PAHO |  |
| 487 | PRY | 2011 | 0.670 | 0.004 | 0.666 | Upper middle income | PAHO |  |
| 488 | PRY | 2012 | 0.610 | 0.004 | 0.606 | Upper middle income | PAHO |  |
| 489 | PRY | 2013 | 0.580 | 0.005 | 0.575 | Upper middle income | PAHO |  |
| 490 | PRY | 2014 | 0.670 | 0.005 | 0.665 | Upper middle income | PAHO |  |
| 491 | PRY | 2015 | 0.570 | 0.006 | 0.564 | Upper middle income | PAHO |  |
| 492 | PRY | 2016 | 0.710 | 0.007 | 0.703 | Upper middle income | PAHO |  |
| 493 | PRY | 2017 | 0.710 | 0.012 | 0.698 | Upper middle income | PAHO |  |
| 494 | PRY | 2018 | 0.770 | 0.013 | 0.757 | Upper middle income | PAHO |  |
| 495 | PER | 2010 | 0.405 | 0.016 | 0.389 | Upper middle income | PAHO |  |
| 496 | PER | 2011 | 0.590 | 0.011 | 0.579 | Upper middle income | PAHO |  |
| 497 | PER | 2012 | 0.690 | 0.016 | 0.674 | Upper middle income | PAHO |  |
| 498 | PER | 2013 | 0.710 | 0.016 | 0.694 | Upper middle income | PAHO |  |
| 499 | PER | 2014 | 0.716 | 0.019 | 0.697 | Upper middle income | PAHO |  |
| 500 | PER | 2015 | 0.680 | 0.022 | 0.658 | Upper middle income | PAHO |  |
| 501 | PER | 2016 | 0.728 | 0.030 | 0.698 | Upper middle income | PAHO |  |
| 502 | PER | 2017 | 0.783 | 0.034 | 0.749 | Upper middle income | PAHO |  |
| 503 | PER | 2018 | 0.904 | 0.040 | 0.864 | Upper middle income | PAHO |  |
| 504 | PRT | 2010 | 1.000 | 0.002 | 0.998 | High income | EURO |  |
| 505 | PRT | 2011 | 1.000 | 0.002 | 0.998 | High income | EURO |  |
| 506 | PRT | 2012 | 1.000 | 0.003 | 0.997 | High income | EURO |  |
| 507 | PRT | 2013 | 1.000 | 0.003 | 0.997 | High income | EURO |  |
| 508 | PRT | 2014 | 1.000 | 0.005 | 0.995 | High income | EURO |  |
| 509 | PRT | 2015 | 1.000 | 0.007 | 0.993 | High income | EURO |  |
| 510 | PRT | 2016 | 1.000 | 0.010 | 0.990 | High income | EURO |  |
| 511 | PRT | 2017 | 1.000 | 0.043 | 0.957 | High income | EURO |  |
| 512 | PRT | 2018 | 1.000 | 0.060 | 0.940 | High income | EURO |  |
| 513 | MDA | 2010 | 0.630 | 0.002 | 0.628 | Lower middle income | EURO |  |
| 514 | MDA | 2011 | 0.620 | 0.004 | 0.616 | Lower middle income | EURO |  |
| 515 | MDA | 2012 | 0.680 | 0.004 | 0.676 | Lower middle income | EURO |  |
| 516 | MDA | 2013 | 0.690 | 0.003 | 0.687 | Lower middle income | EURO |  |
| 517 | MDA | 2014 | 0.690 | 0.003 | 0.687 | Lower middle income | EURO |  |
| 518 | MDA | 2015 | 0.785 | 0.020 | 0.765 | Lower middle income | EURO |  |
| 519 | MDA | 2016 | 0.830 | 0.006 | 0.824 | Lower middle income | EURO |  |
| 520 | MDA | 2017 | 0.880 | 0.006 | 0.874 | Lower middle income | EURO |  |
| 521 | MDA | 2018 | 0.760 | 0.008 | 0.752 | Lower middle income | EURO |  |
| 522 | ROU | 2010 | 1.000 | 0.006 | 0.994 | Upper middle income | EURO |  |
| 523 | ROU | 2011 | 1.000 | 0.006 | 0.994 | Upper middle income | EURO |  |
| 524 | ROU | 2012 | 1.000 | 0.008 | 0.992 | Upper middle income | EURO |  |
| 525 | ROU | 2013 | 1.000 | 0.009 | 0.991 | Upper middle income | EURO |  |
| 526 | ROU | 2014 | 1.000 | 0.010 | 0.990 | Upper middle income | EURO |  |
| 527 | ROU | 2015 | 1.000 | 0.016 | 0.984 | Upper middle income | EURO |  |
| 528 | ROU | 2016 | 1.000 | 0.011 | 0.989 | Upper middle income | EURO |  |
| 529 | ROU | 2017 | 1.000 | 0.016 | 0.984 | Upper middle income | EURO |  |
| 530 | ROU | 2018 | 1.000 | 0.015 | 0.985 | Upper middle income | EURO |  |
| 531 | RWA | 2010 | 0.610 | 0.003 | 0.607 | Low income | AFRO |  |
| 532 | RWA | 2011 | 0.960 | 0.002 | 0.958 | Low income | AFRO |  |
| 533 | RWA | 2012 | 0.840 | 0.003 | 0.837 | Low income | AFRO |  |
| 534 | RWA | 2013 | 0.890 | 0.004 | 0.886 | Low income | AFRO |  |
| 535 | RWA | 2014 | 0.950 | 0.005 | 0.945 | Low income | AFRO |  |
| 536 | RWA | 2015 | 1.000 | 0.011 | 0.989 | Low income | AFRO |  |
| 537 | RWA | 2016 | 0.962 | 0.026 | 0.936 | Low income | AFRO |  |
| 538 | RWA | 2017 | 0.969 | 0.032 | 0.937 | Low income | AFRO |  |
| 539 | RWA | 2018 | 1.000 | 0.038 | 0.962 | Low income | AFRO |  |
| 540 | SEN | 2010 | 0.335 | 0.006 | 0.329 | Lower middle income | AFRO |  |
| 541 | SEN | 2011 | 0.354 | 0.008 | 0.346 | Lower middle income | AFRO |  |
| 542 | SEN | 2012 | 0.353 | 0.013 | 0.339 | Lower middle income | AFRO |  |
| 543 | SEN | 2013 | 0.493 | 0.013 | 0.480 | Lower middle income | AFRO |  |
| 544 | SEN | 2014 | 0.469 | 0.020 | 0.448 | Lower middle income | AFRO |  |
| 545 | SEN | 2015 | 0.578 | 0.019 | 0.558 | Lower middle income | AFRO |  |
| 546 | SEN | 2016 | 0.707 | 0.033 | 0.674 | Lower middle income | AFRO |  |
| 547 | SEN | 2017 | 0.681 | 0.024 | 0.657 | Lower middle income | AFRO |  |
| 548 | SEN | 2018 | 0.780 | 0.029 | 0.750 | Lower middle income | AFRO |  |
| 549 | SLE | 2010 | 0.280 | 0.001 | 0.279 | Low income | AFRO |  |
| 550 | SLE | 2011 | 0.350 | 0.002 | 0.348 | Low income | AFRO |  |
| 551 | SLE | 2012 | 0.440 | 0.002 | 0.438 | Low income | AFRO |  |
| 552 | SLE | 2013 | 0.380 | 0.003 | 0.377 | Low income | AFRO |  |
| 553 | SLE | 2014 | 0.370 | 0.002 | 0.368 | Low income | AFRO |  |
| 554 | SLE | 2015 | 0.493 | 0.010 | 0.483 | Low income | AFRO |  |
| 555 | SLE | 2016 | 0.520 | 0.004 | 0.516 | Low income | AFRO |  |
| 556 | SLE | 2017 | 0.540 | 0.008 | 0.532 | Low income | AFRO |  |
| 557 | SLE | 2018 | 0.620 | 0.010 | 0.610 | Low income | AFRO |  |
| 558 | ZAF | 2010 | 0.710 | 0.001 | 0.709 | Upper middle income | AFRO |  |
| 559 | ZAF | 2011 | 0.880 | 0.001 | 0.879 | Upper middle income | AFRO |  |
| 560 | ZAF | 2012 | 0.900 | 0.001 | 0.899 | Upper middle income | AFRO |  |
| 561 | ZAF | 2013 | 0.960 | 0.002 | 0.958 | Upper middle income | AFRO |  |
| 562 | ZAF | 2014 | 0.970 | 0.002 | 0.968 | Upper middle income | AFRO |  |
| 563 | ZAF | 2015 | 0.970 | 0.002 | 0.968 | Upper middle income | AFRO |  |
| 564 | ZAF | 2016 | 0.980 | 0.002 | 0.978 | Upper middle income | AFRO |  |
| 565 | ZAF | 2017 | 0.930 | 0.002 | 0.928 | Upper middle income | AFRO |  |
| 566 | ZAF | 2018 | 0.960 | 0.003 | 0.957 | Upper middle income | AFRO |  |
| 567 | ESP | 2010 | 1.000 | 0.003 | 0.997 | High income | EURO |  |
| 568 | ESP | 2011 | 1.000 | 0.004 | 0.996 | High income | EURO |  |
| 569 | ESP | 2012 | 1.000 | 0.005 | 0.995 | High income | EURO |  |
| 570 | ESP | 2013 | 1.000 | 0.006 | 0.994 | High income | EURO |  |
| 571 | ESP | 2014 | 1.000 | 0.006 | 0.994 | High income | EURO |  |
| 572 | ESP | 2015 | 1.000 | 0.010 | 0.990 | High income | EURO |  |
| 573 | ESP | 2016 | 1.000 | 0.014 | 0.986 | High income | EURO |  |
| 574 | ESP | 2017 | 1.000 | 0.023 | 0.977 | High income | EURO |  |
| 575 | ESP | 2018 | 1.000 | 0.029 | 0.971 | High income | EURO |  |
| 576 | SDN | 2010 | 0.074 | 0.004 | 0.070 | Lower middle income | EMRO |  |
| 577 | SDN | 2011 | 0.078 | 0.004 | 0.074 | Lower middle income | EMRO |  |
| 578 | SDN | 2012 | 0.082 | 0.004 | 0.078 | Lower middle income | EMRO |  |
| 579 | SDN | 2013 | 0.095 | 0.005 | 0.090 | Lower middle income | EMRO |  |
| 580 | SUR | 2010 | 0.620 | 0.002 | 0.618 | Upper middle income | PAHO |  |
| 581 | SUR | 2011 | 0.910 | 0.002 | 0.908 | Upper middle income | PAHO |  |
| 582 | SUR | 2012 | 0.900 | 0.003 | 0.897 | Upper middle income | PAHO |  |
| 583 | SUR | 2013 | 0.900 | 0.003 | 0.897 | Upper middle income | PAHO |  |
| 584 | SUR | 2014 | 0.940 | 0.003 | 0.937 | Upper middle income | PAHO |  |
| 585 | SUR | 2015 | 1.000 | 0.003 | 0.997 | Upper middle income | PAHO |  |
| 586 | SUR | 2016 | 0.890 | 0.004 | 0.886 | Upper middle income | PAHO |  |
| 587 | SUR | 2017 | 0.780 | 0.005 | 0.775 | Upper middle income | PAHO |  |
| 588 | SUR | 2018 | 0.760 | 0.011 | 0.749 | Upper middle income | PAHO |  |
| 589 | TJK | 2010 | 0.430 | 0.003 | 0.427 | Low income | EURO |  |
| 590 | TJK | 2011 | 0.545 | 0.013 | 0.533 | Low income | EURO |  |
| 591 | TJK | 2012 | 0.650 | 0.004 | 0.646 | Low income | EURO |  |
| 592 | TJK | 2013 | 0.720 | 0.005 | 0.715 | Low income | EURO |  |
| 593 | TJK | 2014 | 1.000 | 0.024 | 0.976 | Low income | EURO |  |
| 594 | TJK | 2015 | 0.985 | 0.022 | 0.963 | Low income | EURO |  |
| 595 | TJK | 2016 | 0.960 | 0.007 | 0.953 | Low income | EURO |  |
| 596 | TJK | 2017 | 0.810 | 0.009 | 0.801 | Low income | EURO |  |
| 597 | TJK | 2018 | 0.980 | 0.011 | 0.969 | Low income | EURO |  |
| 598 | THA | 2010 | 0.940 | 0.003 | 0.937 | Upper middle income | SEARO |  |
| 599 | THA | 2011 | 0.940 | 0.003 | 0.937 | Upper middle income | SEARO |  |
| 600 | THA | 2012 | 0.940 | 0.004 | 0.936 | Upper middle income | SEARO |  |
| 601 | THA | 2013 | 0.940 | 0.004 | 0.936 | Upper middle income | SEARO |  |
| 602 | THA | 2014 | 0.950 | 0.005 | 0.945 | Upper middle income | SEARO |  |
| 603 | THA | 2015 | 0.960 | 0.006 | 0.954 | Upper middle income | SEARO |  |
| 604 | THA | 2016 | 0.970 | 0.010 | 0.960 | Upper middle income | SEARO |  |
| 605 | THA | 2017 | 0.973 | 0.014 | 0.958 | Upper middle income | SEARO |  |
| 606 | THA | 2018 | 0.997 | 0.014 | 0.983 | Upper middle income | SEARO |  |
| 607 | TGO | 2010 | 0.311 | 0.008 | 0.303 | Low income | AFRO |  |
| 608 | TGO | 2011 | 0.630 | 0.003 | 0.627 | Low income | AFRO |  |
| 609 | TGO | 2012 | 0.670 | 0.005 | 0.665 | Low income | AFRO |  |
| 610 | TGO | 2013 | 0.700 | 0.003 | 0.697 | Low income | AFRO |  |
| 611 | TGO | 2014 | 0.710 | 0.004 | 0.706 | Low income | AFRO |  |
| 612 | TGO | 2015 | 0.760 | 0.010 | 0.750 | Low income | AFRO |  |
| 613 | TGO | 2016 | 0.820 | 0.009 | 0.811 | Low income | AFRO |  |
| 614 | TGO | 2017 | 0.695 | 0.017 | 0.678 | Low income | AFRO |  |
| 615 | TGO | 2018 | 0.773 | 0.016 | 0.757 | Low income | AFRO |  |
| 616 | UKR | 2010 | 0.870 | 0.006 | 0.864 | Lower middle income | EURO |  |
| 617 | UKR | 2011 | 0.910 | 0.006 | 0.904 | Lower middle income | EURO |  |
| 618 | UKR | 2012 | 0.910 | 0.004 | 0.906 | Lower middle income | EURO |  |
| 619 | UKR | 2013 | 0.910 | 0.004 | 0.906 | Lower middle income | EURO |  |
| 620 | UKR | 2014 | 0.920 | 0.004 | 0.916 | Lower middle income | EURO |  |
| 621 | UKR | 2015 | 0.910 | 0.004 | 0.906 | Lower middle income | EURO |  |
| 622 | UKR | 2016 | 0.910 | 0.004 | 0.906 | Lower middle income | EURO |  |
| 623 | UKR | 2017 | 0.920 | 0.011 | 0.909 | Lower middle income | EURO |  |
| 624 | UKR | 2018 | 0.920 | 0.018 | 0.902 | Lower middle income | EURO |  |
| 625 | UZB | 2011 | 0.261 | 0.006 | 0.255 | Lower middle income | EURO |  |
| 626 | UZB | 2012 | 0.657 | 0.016 | 0.641 | Lower middle income | EURO |  |
| 627 | UZB | 2014 | 0.781 | 0.020 | 0.761 | Lower middle income | EURO |  |
| 628 | UZB | 2016 | 0.838 | 0.018 | 0.820 | Lower middle income | EURO |  |
| 629 | UZB | 2017 | 0.741 | 0.023 | 0.718 | Lower middle income | EURO |  |
| 630 | UZB | 2018 | 0.852 | 0.025 | 0.827 | Lower middle income | EURO |  |
| 631 | VNM | 2010 | 0.487 | 0.011 | 0.476 | Lower middle income | WPRO |  |
| 632 | VNM | 2011 | 0.408 | 0.012 | 0.396 | Lower middle income | WPRO |  |
| 633 | VNM | 2012 | 0.493 | 0.023 | 0.470 | Lower middle income | WPRO |  |
| 634 | VNM | 2013 | 0.516 | 0.020 | 0.495 | Lower middle income | WPRO |  |
| 635 | VNM | 2014 | 0.644 | 0.022 | 0.622 | Lower middle income | WPRO |  |
| 636 | VNM | 2015 | 0.705 | 0.034 | 0.671 | Lower middle income | WPRO |  |
| 637 | VNM | 2016 | 0.796 | 0.035 | 0.760 | Lower middle income | WPRO |  |
| 638 | VNM | 2017 | 0.772 | 0.033 | 0.739 | Lower middle income | WPRO |  |
| 639 | VNM | 2018 | 0.812 | 0.035 | 0.777 | Lower middle income | WPRO |  |
| 640 | ZMB | 2010 | 0.670 | 0.002 | 0.668 | Lower middle income | AFRO |  |
| 641 | ZMB | 2011 | 0.700 | 0.002 | 0.698 | Lower middle income | AFRO |  |
| 642 | ZMB | 2012 | 0.730 | 0.002 | 0.728 | Lower middle income | AFRO |  |
| 643 | ZMB | 2013 | 0.770 | 0.003 | 0.767 | Lower middle income | AFRO |  |
| 644 | ZMB | 2014 | 0.820 | 0.006 | 0.814 | Lower middle income | AFRO |  |
| 645 | ZMB | 2015 | 0.860 | 0.011 | 0.849 | Lower middle income | AFRO |  |
| 646 | ZMB | 2016 | 0.852 | 0.016 | 0.836 | Lower middle income | AFRO |  |
| 647 | ZMB | 2017 | 0.900 | 0.016 | 0.884 | Lower middle income | AFRO |  |
| 648 | ZMB | 2018 | 0.923 | 0.036 | 0.886 | Lower middle income | AFRO |  |
| 649 | ZWE | 2010 | 0.350 | 0.012 | 0.338 | Lower middle income | AFRO |  |
| 650 | ZWE | 2011 | 0.526 | 0.008 | 0.518 | Lower middle income | AFRO |  |
| 651 | ZWE | 2012 | 0.790 | 0.002 | 0.788 | Lower middle income | AFRO |  |
| 652 | ZWE | 2013 | 0.790 | 0.004 | 0.786 | Lower middle income | AFRO |  |
| 653 | ZWE | 2014 | 0.810 | 0.007 | 0.803 | Lower middle income | AFRO |  |
| 654 | ZWE | 2015 | 0.810 | 0.015 | 0.795 | Lower middle income | AFRO |  |
| 655 | ZWE | 2016 | 0.872 | 0.022 | 0.850 | Lower middle income | AFRO |  |
| 656 | ZWE | 2017 | 0.927 | 0.034 | 0.892 | Lower middle income | AFRO |  |
| 657 | ZWE | 2018 | 0.994 | 0.040 | 0.954 | Lower middle income | AFRO |  |

*Notes:* First DEA column stands for vector of coefficients in the truncated regression of the reciprocal of efficiency score on environmental variables. Robust DEA adjusted stands for the vector of robust coefficients in the truncated regression of reciprocal of efficiency score on independent variables (after the second loop).

**Table D.** Correlation between efficiency scores and our independent variables

| **VARIABLES** |  | | **Pearson correlation** |
| --- | --- | --- | --- |
| Rule of Law |  |  | 0.409 |
| Antenatal Care Coverage | |  | 0.339 |
| GNI per capita in USD | |  | 0.313 |
| CHE as % of GDP | |  | 0.192 |
| CHE per capita in USD | |  | 0.324 |
| Population per KM^2^ | |  | -0.001 |
| Human Development Index (HDI) | | | 0.424 |
| HIV prevalence |  |  | 0.189 |
| OOPE as % of the total HIV spending | | | -0.159 |
| DAHS per total HIV spending ratio | | | -0.248 |
| Nurses per 10k population | | | 0.351 |
| Total density health posts | |  | 0.067 |
| GS per total HIV spending ratio | | | 0.097 |
| External expenditure as % of CHE | | | -0.180 |

*Notes*: CHE: Catastrophic Health Expenditure, HDI: Human Development Index, GNI: Gross National Income, GS: Government spending, OOPE: Out-of-pocket expenditure, USD: United States Dollars, DAHS: Development assistance for health spending

**Table E.** Univariate analysis for the truncated regression model for the reciprocal of the efficiency score

| **VARIABLES** |  | | **COEFF** | **SE** | **P-value** |
| --- | --- | --- | --- | --- | --- |
| Rule of Law |  |  | -29.29 | 8.70 | 0.001 |
| Antenatal Care Coverage | |  | -1.52 | 0.90 | 0.089 |
| GNI per capita in USD | |  | 0.00 | 0.00 | 0.017 |
| CHE as % of GDP | |  | -322.22 | 14.82 | 0.000 |
| CHE per capita in USD | |  | -0.08 | 0.04 | 0.020 |
| Population per KM^2^ | |  | 0.05 | 0.02 | 0.011 |
| Human Development Index (HDI) | | | -1.36 | 0.48 | 0.005 |
| HIV prevalence |  |  | -2.95 | 1.23 | 0.017 |
| OOPE as % of the total HIV spending | | | 0.18 | 0.10 | 0.072 |
| DAHS per total HIV spending ratio | | | 0.27 | 0.09 | 0.003 |
| Nurses per 10k | | | -1.46 | 0.64 | 0.022 |
| Total density health posts | |  | -0.26 | 0.13 | 0.04 |
| GS per total HIV spending ratio | | | -2.02 | 1.04 | 0.05 |
| External expenditure as % of CHE | | | 0.18 | 0.08 | 0.04 |

*Notes*: CHE: Catastrophic Health Expenditure, HDI: Human Development Index, GNI: Gross National Income, GS: Government spending, OOPE: Out-of-pocket expenditure, USD: United States Dollars, DAHS: Development assistance for health spending

**Table F.** Variance Inflator Factor estimates for the core adjusted multivariate model

Variance Inflator Factor (VIF) for our main covariates

| Variable | VIF | 1/VIF |
| --- | --- | --- |
| GNI per capita | 7.14 | 0.14011 |
| CHE per capita | 6.67 | 0.1499 |
| HDI | 5.85 | 0.17106 |
| Antenatal care coverage | 4.35 | 0.22963 |
| CHE as a % of the GDP | 2.02 | 0.49563 |
| Rule of law | 1.82 | 0.55052 |
| HIV prevalence | 1.4 | 0.71654 |
| OOPE as % of the total HIV spending | 1.33 | 0.75103 |
| Pop per km | 1.15 | 0.87266 |
| DAHS per total HIV spending ratio | 1.10 | 0.90638 |
| Mean VIF | 3.28 |  |

*Notes*: CHE: Catastrophic Health Expenditure, HDI: Human Development Index, GNI: Gross National Income. OOPE: Out-of-pocket expenditure, DAHS: Development assistance for health spending.

**Figure A.** Univariate relationship between efficiency scores and the independent variables

**(a)**


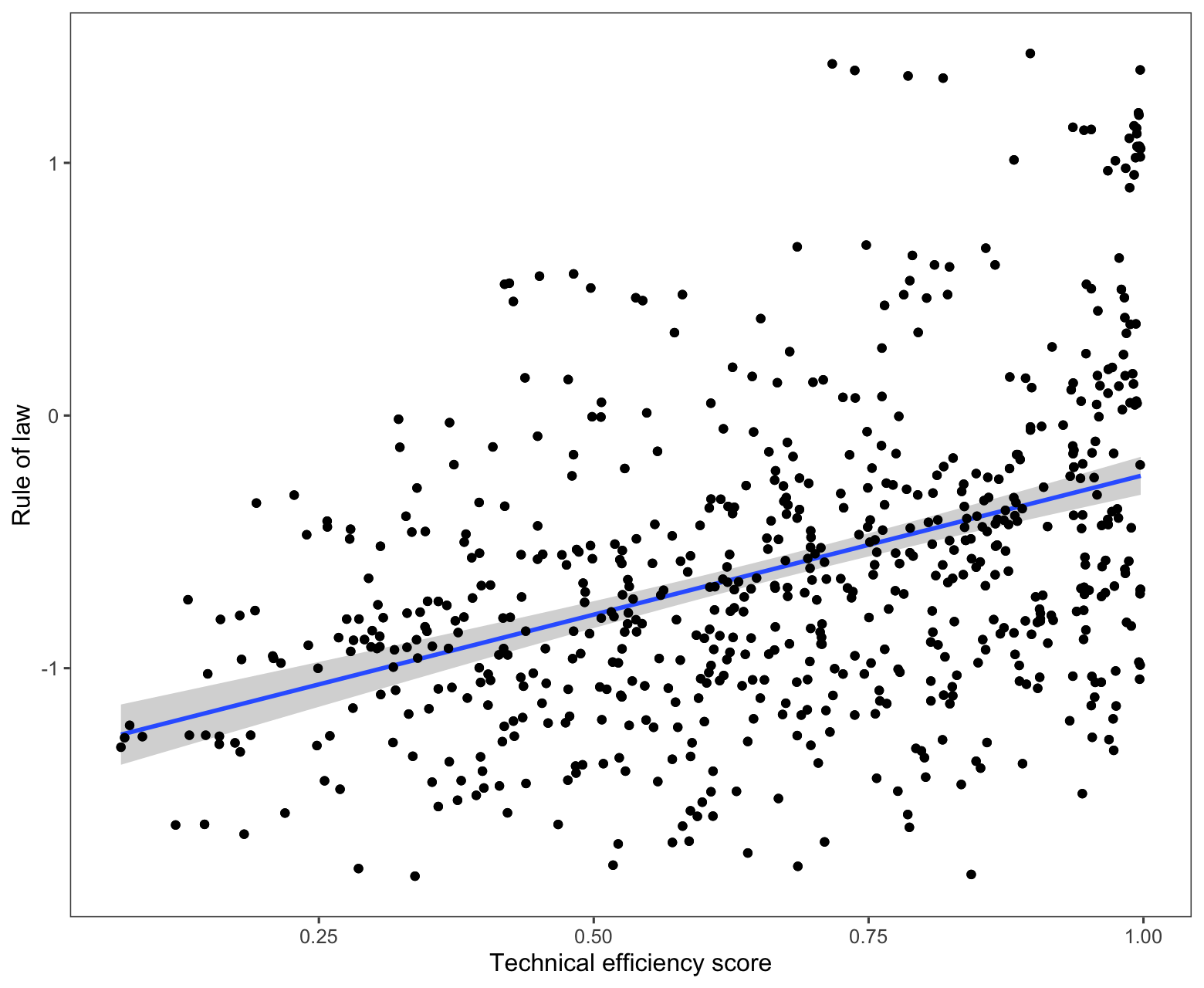


**(b)**

**
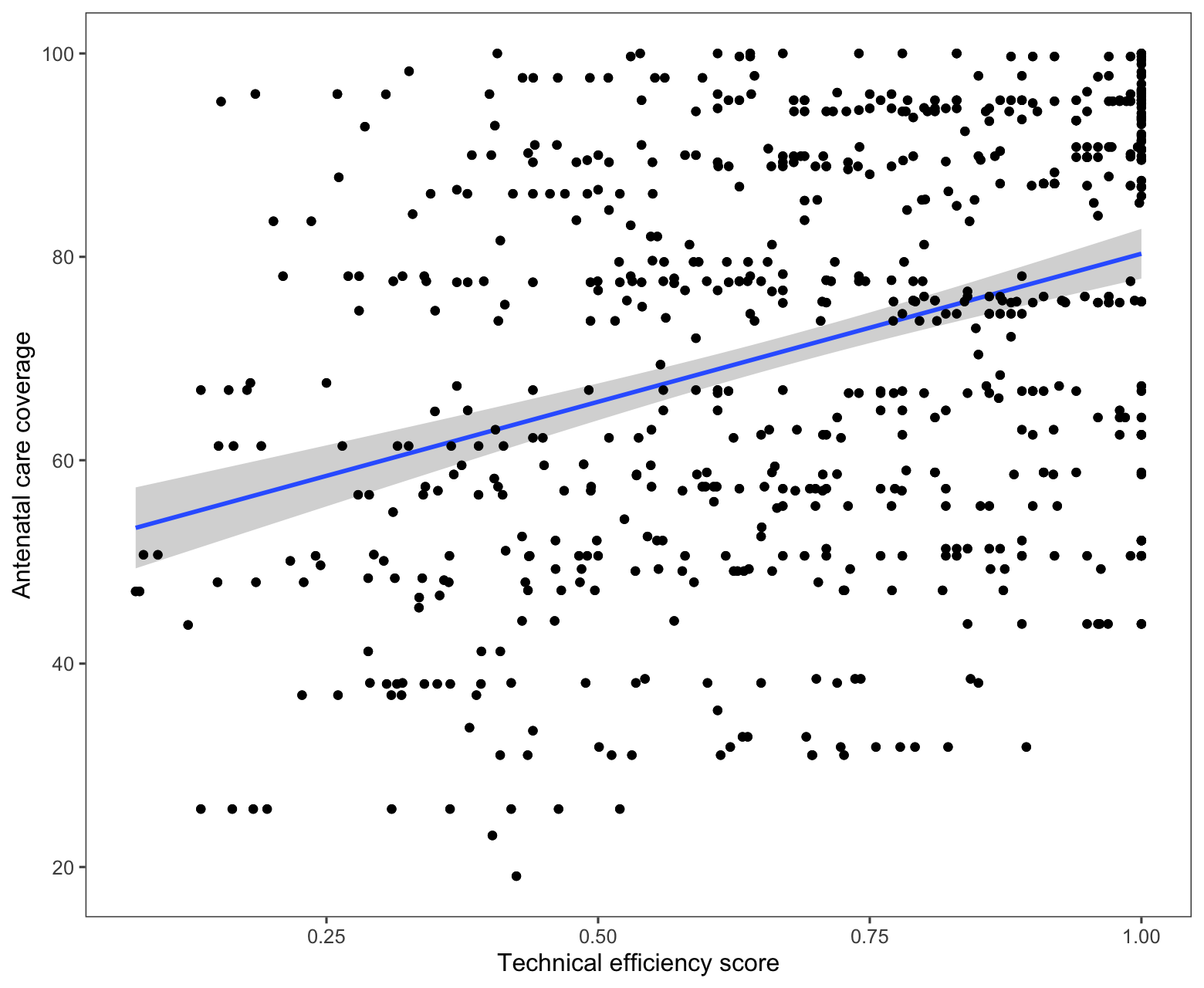
**

**(c)**

**
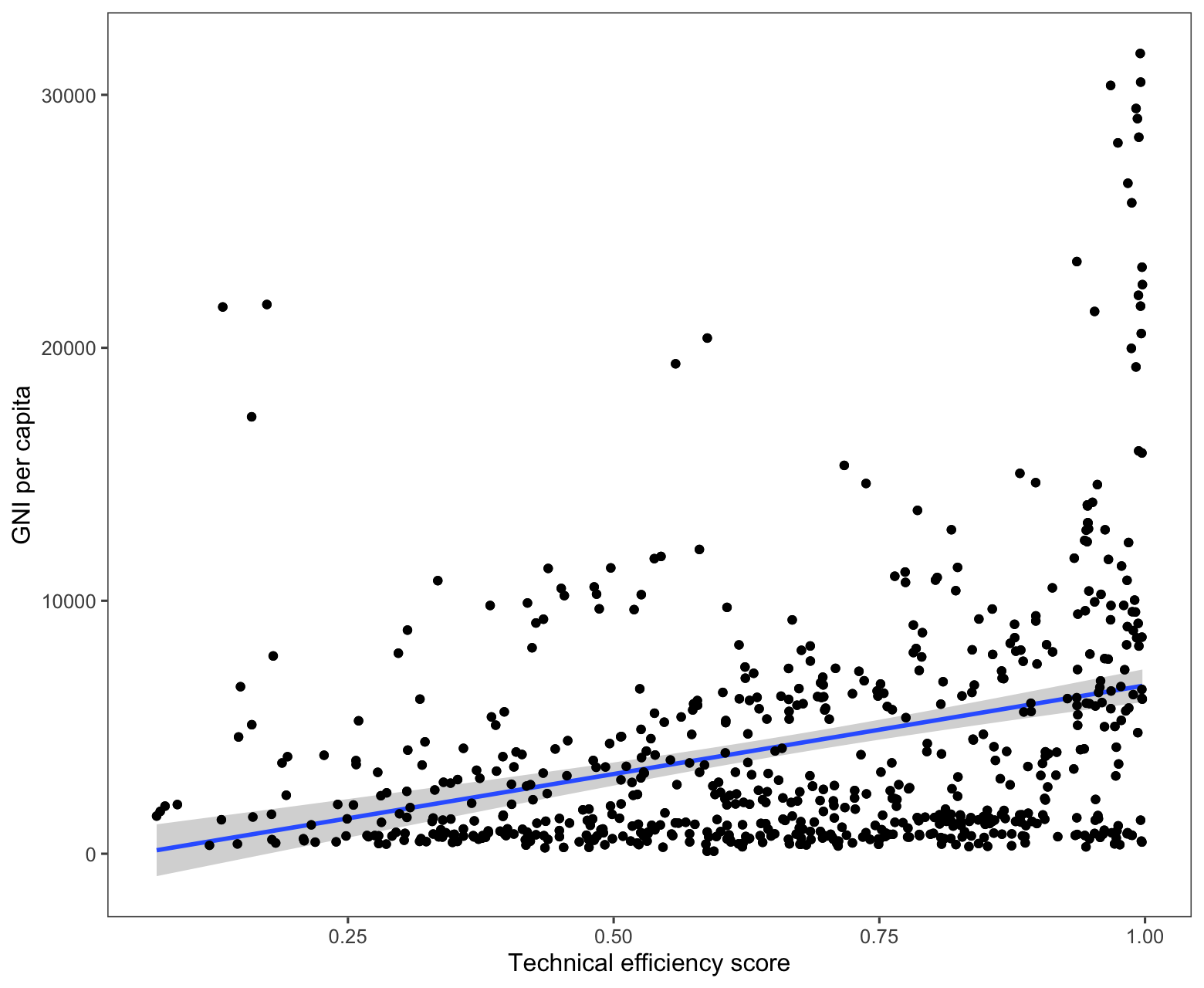
**

**(d)**


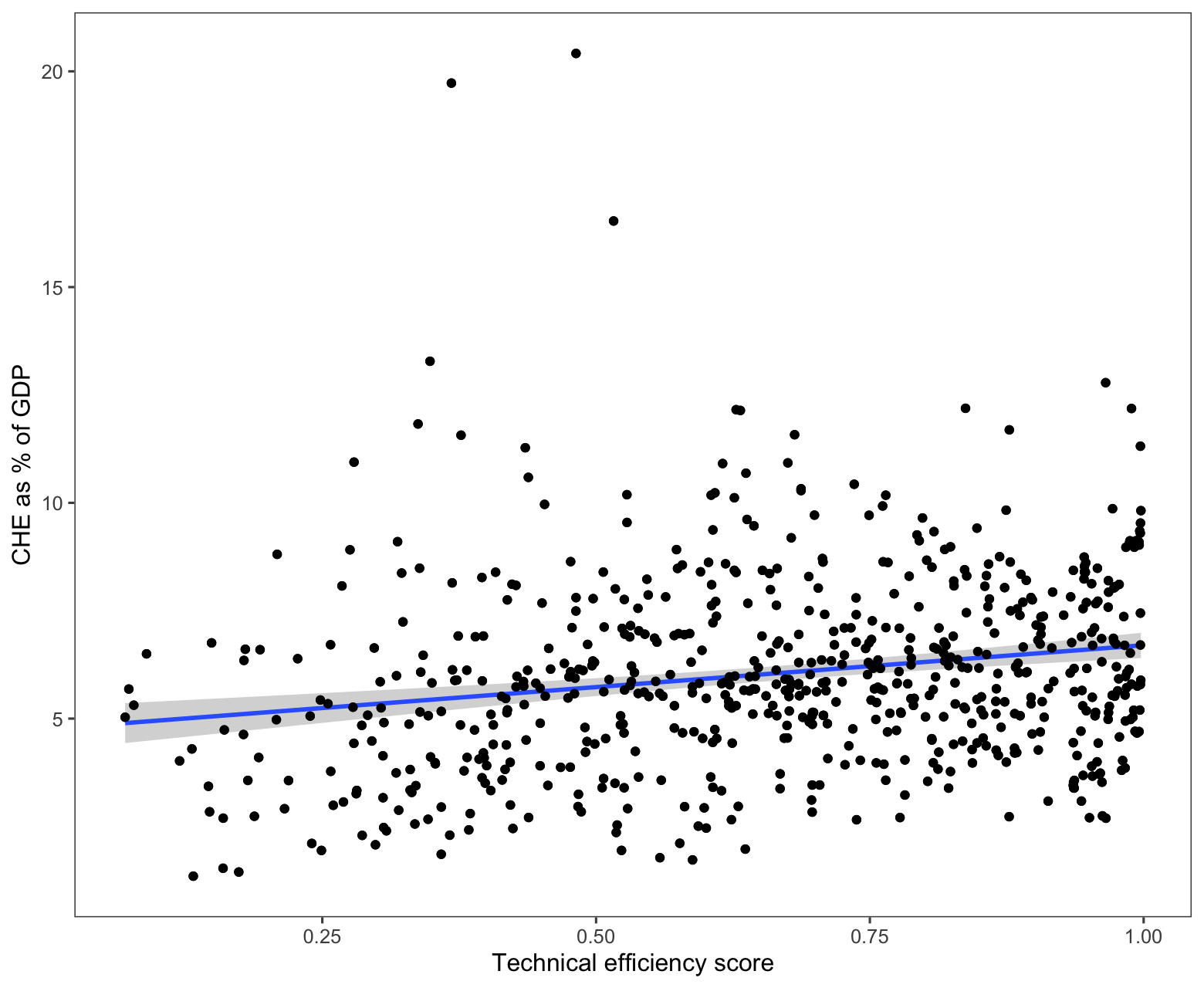


**(e)**


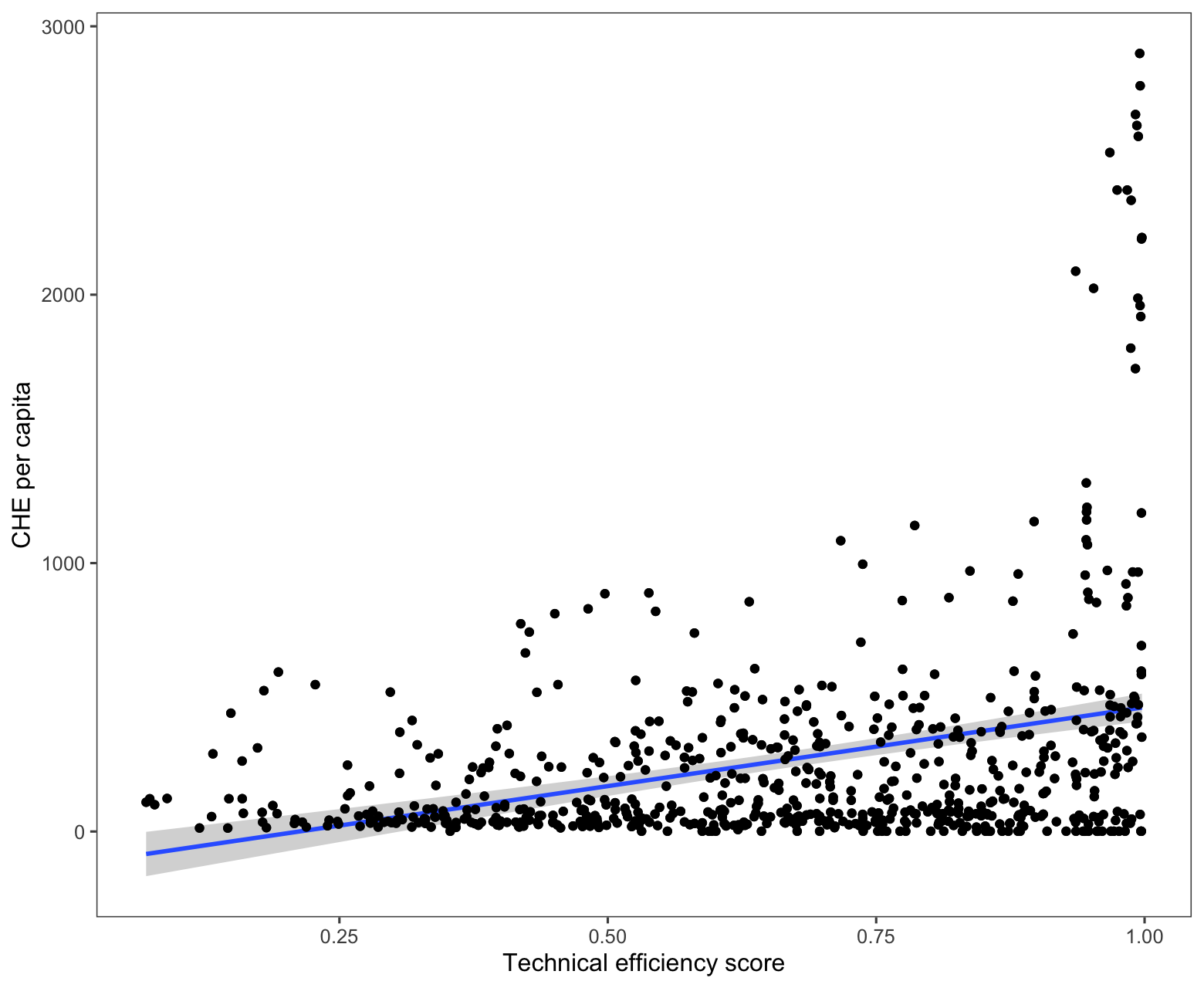


**(f)**


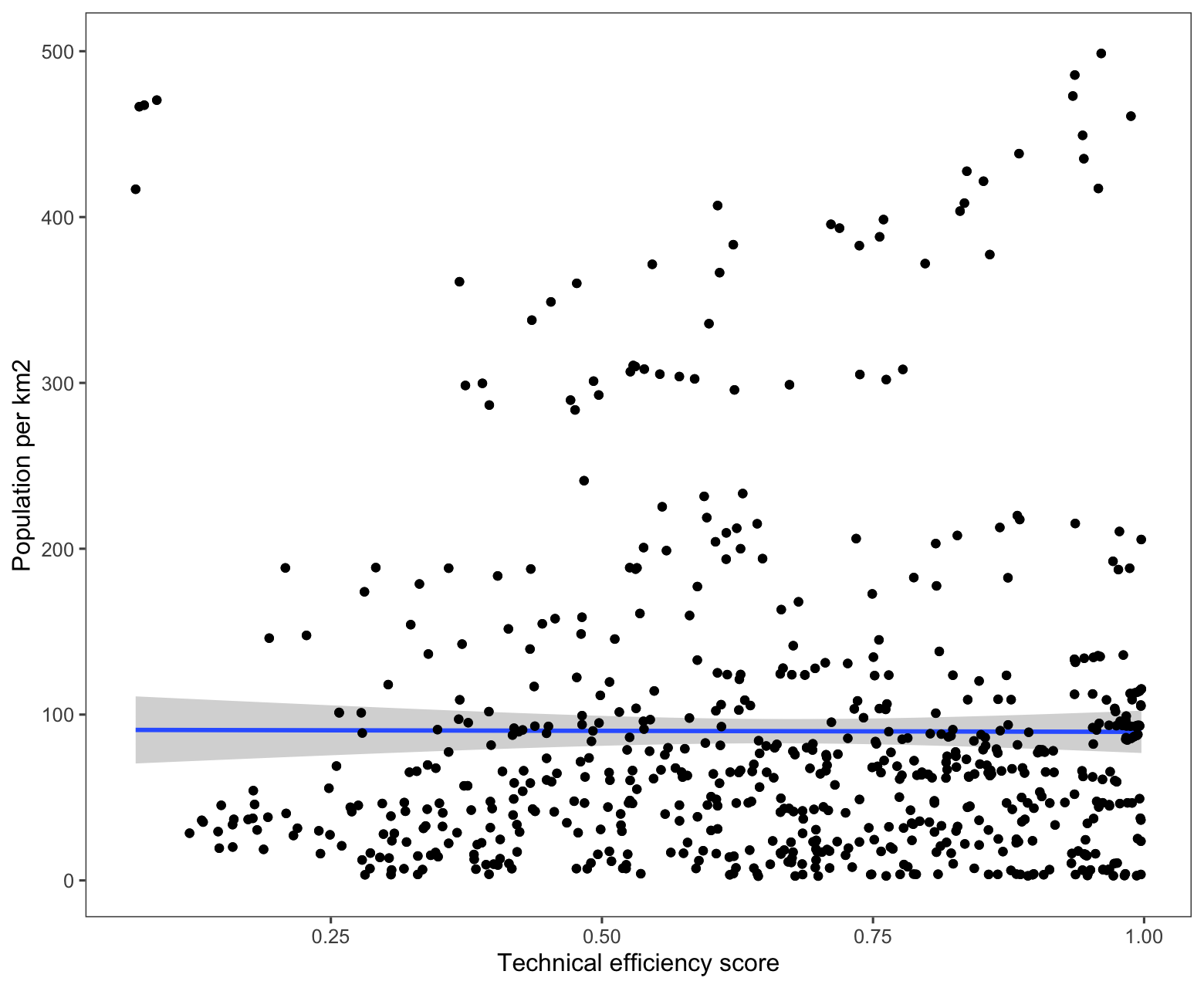


**(g)**

**
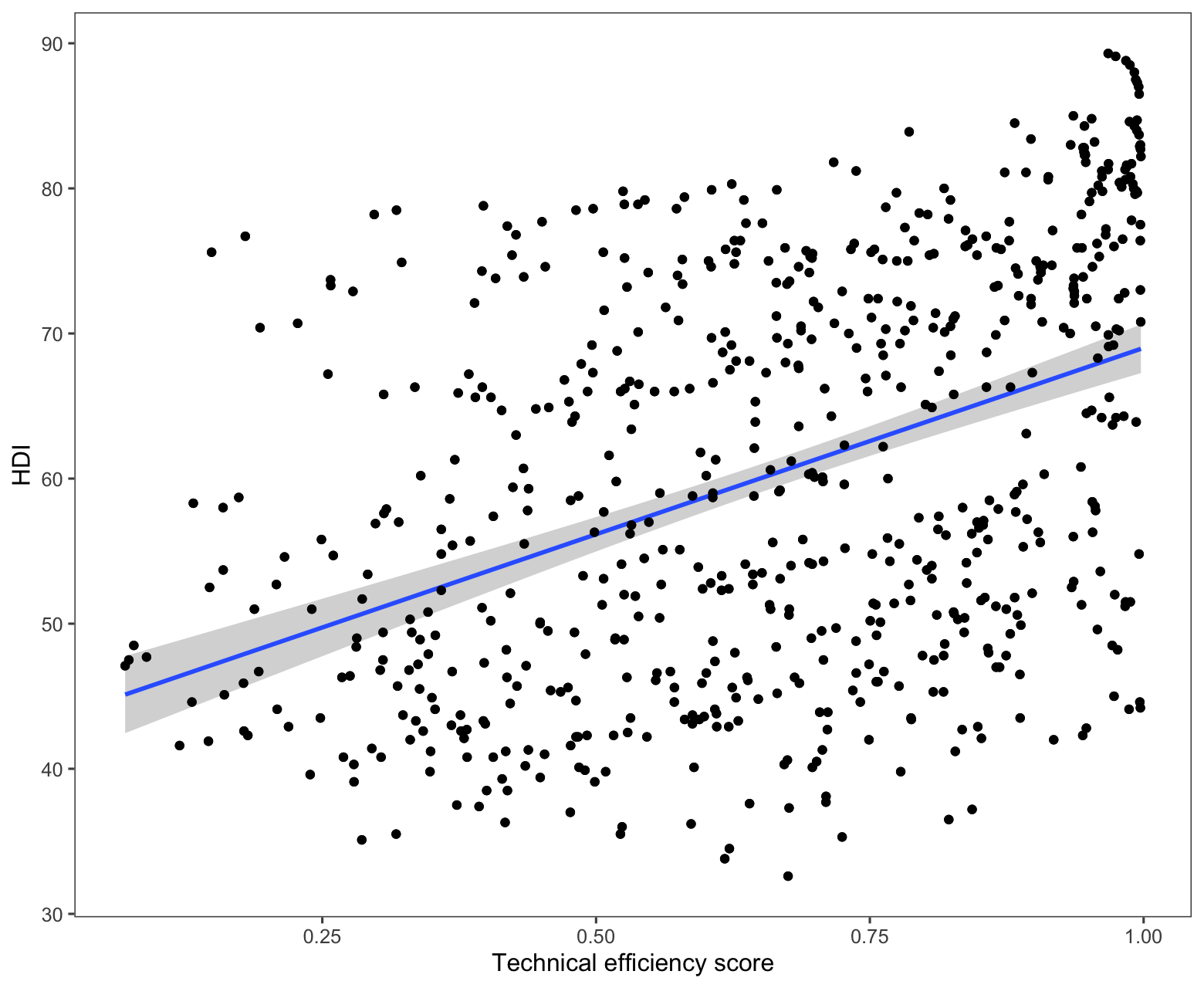
**

**(h)**

**
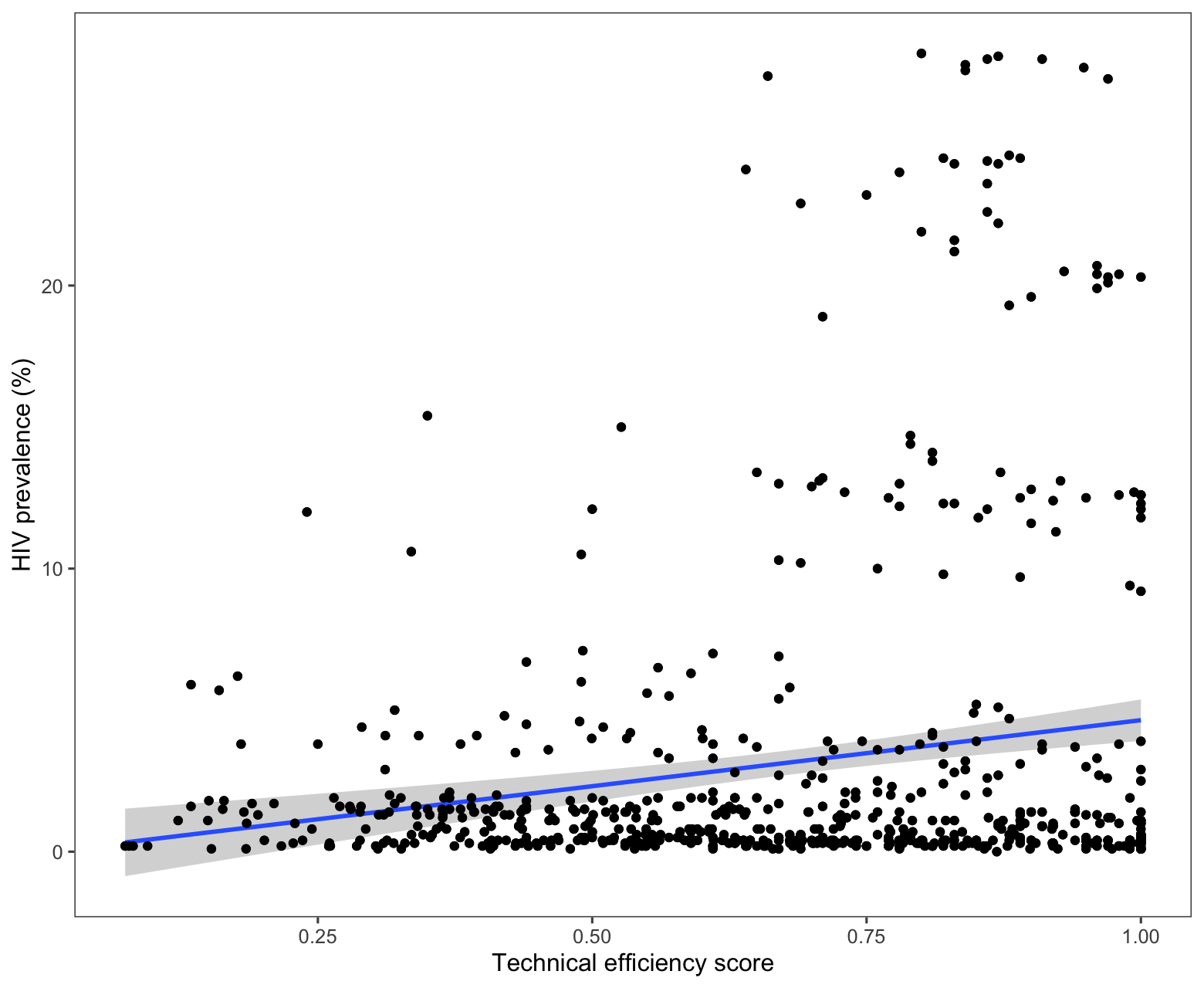
**

**(i)**

**
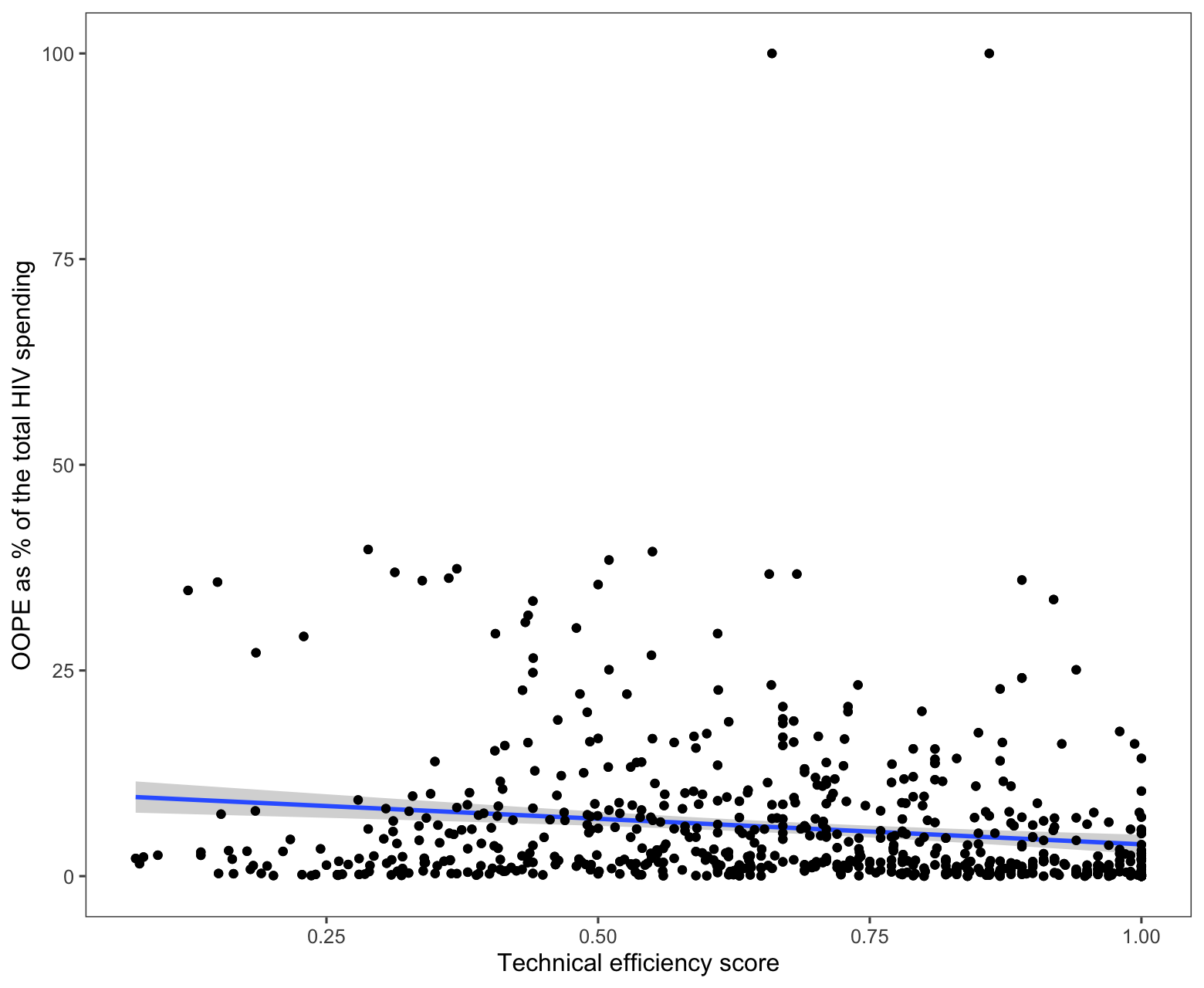
**

**(j)**

**
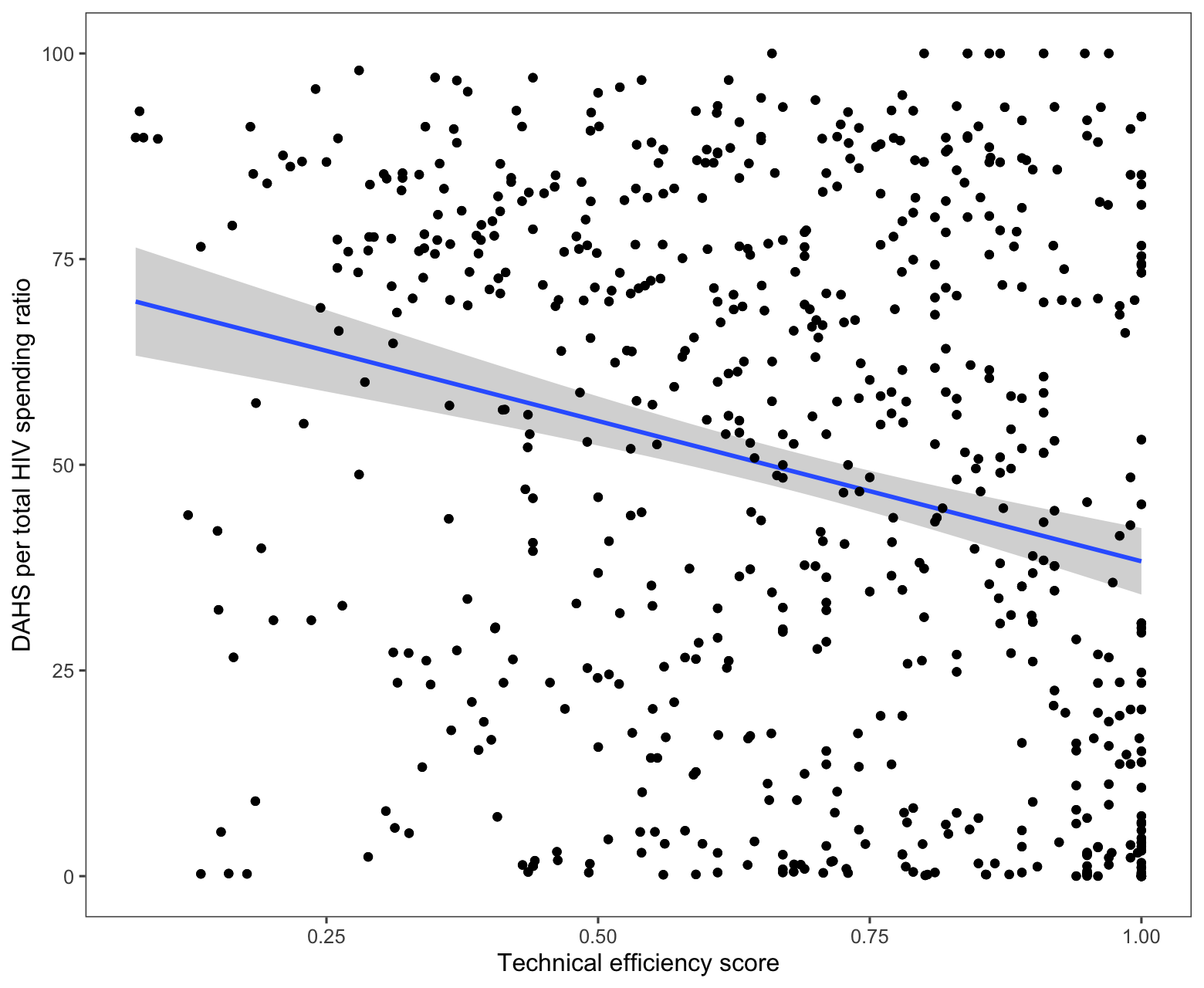
**

**(k)**

**
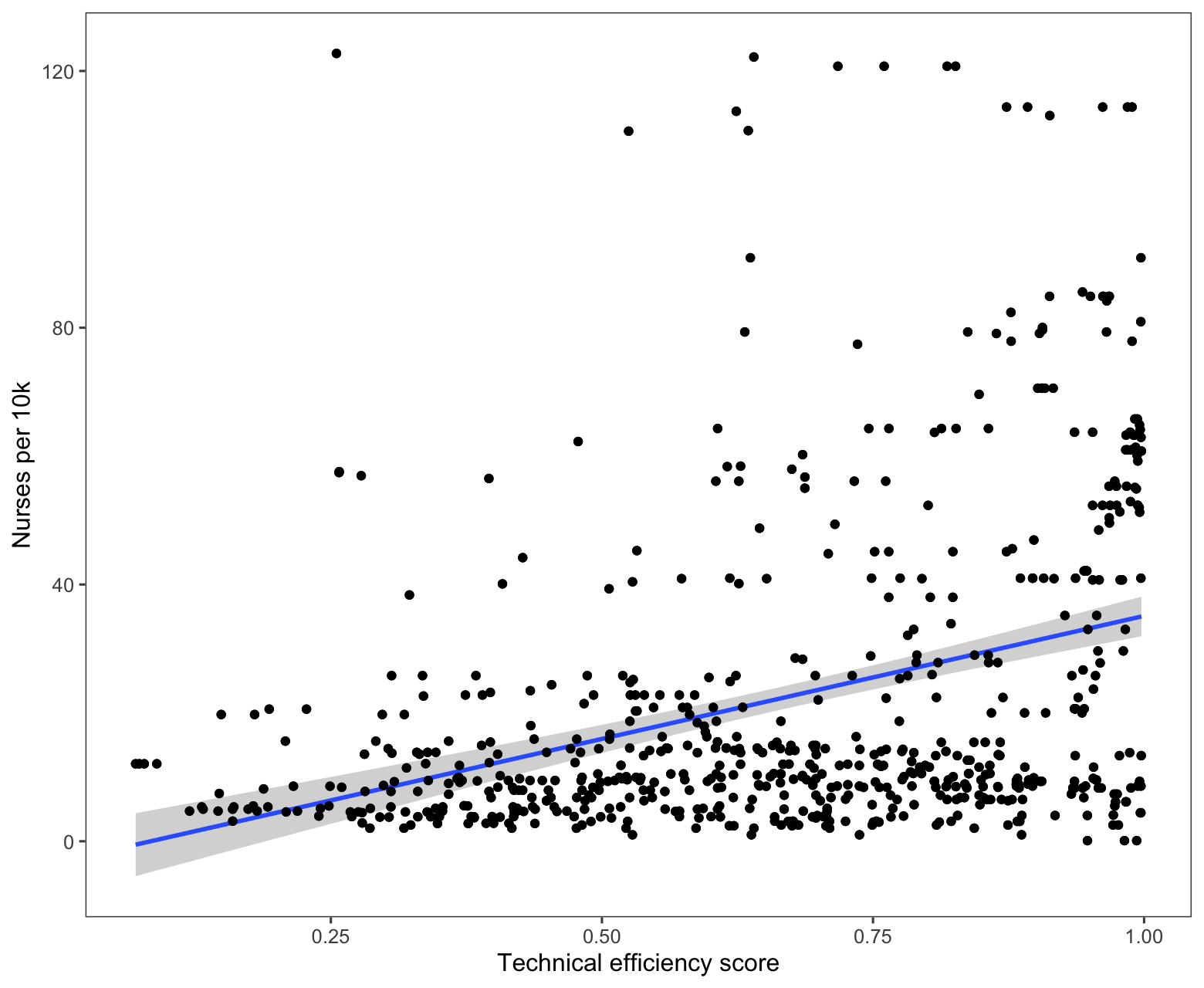
**

**(l)**

**
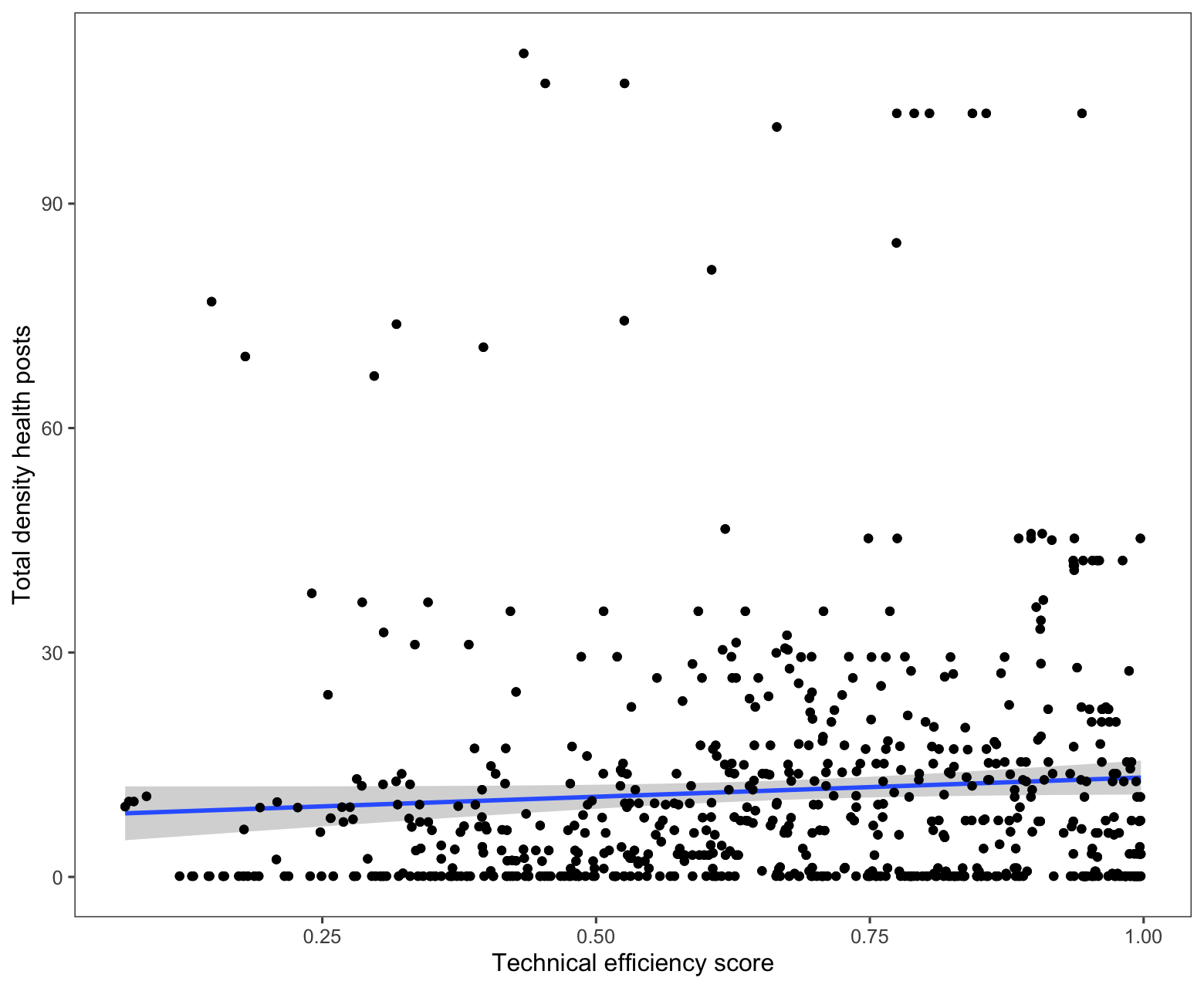
**

**(m)**

**
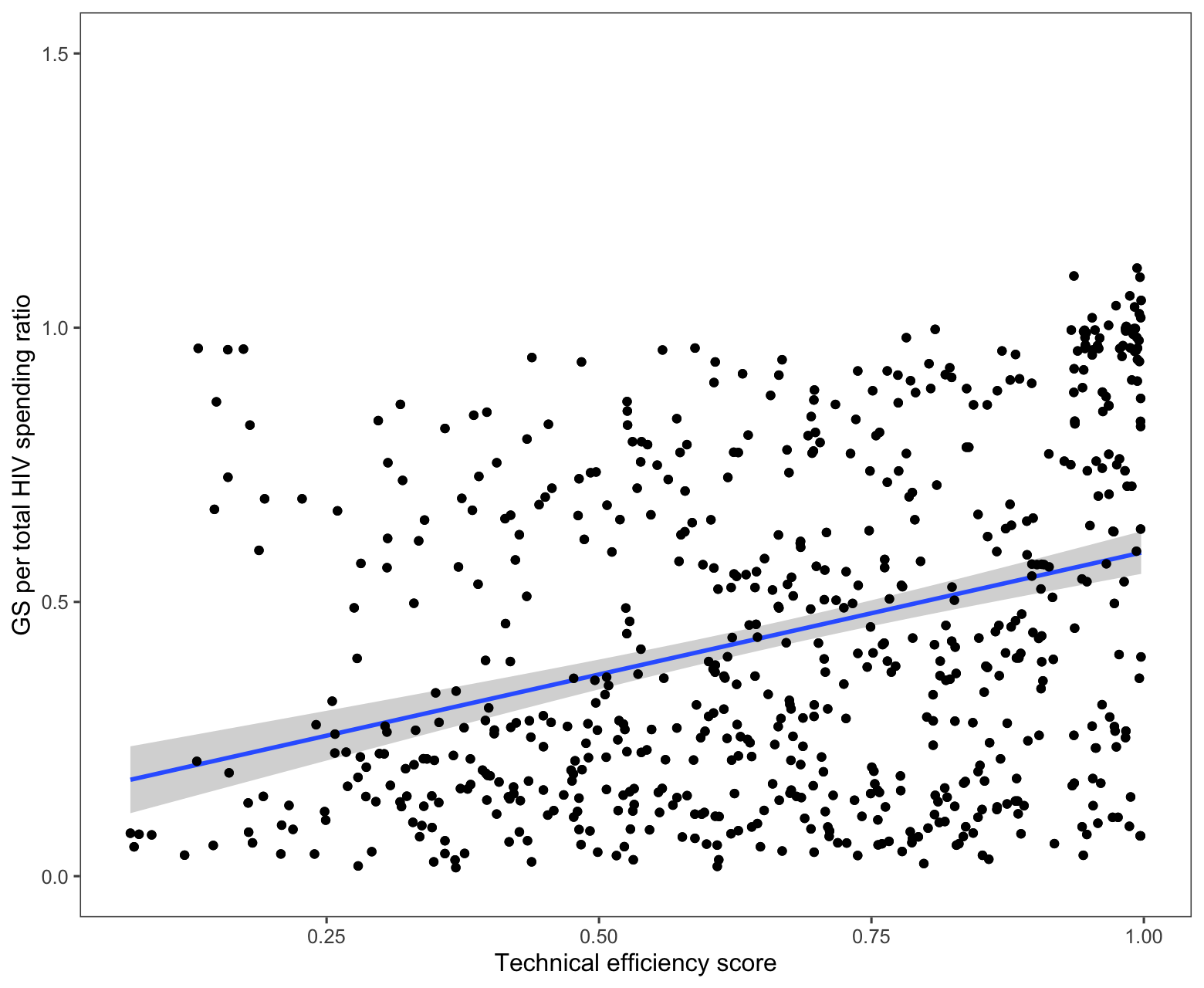
**

**(n)**

**
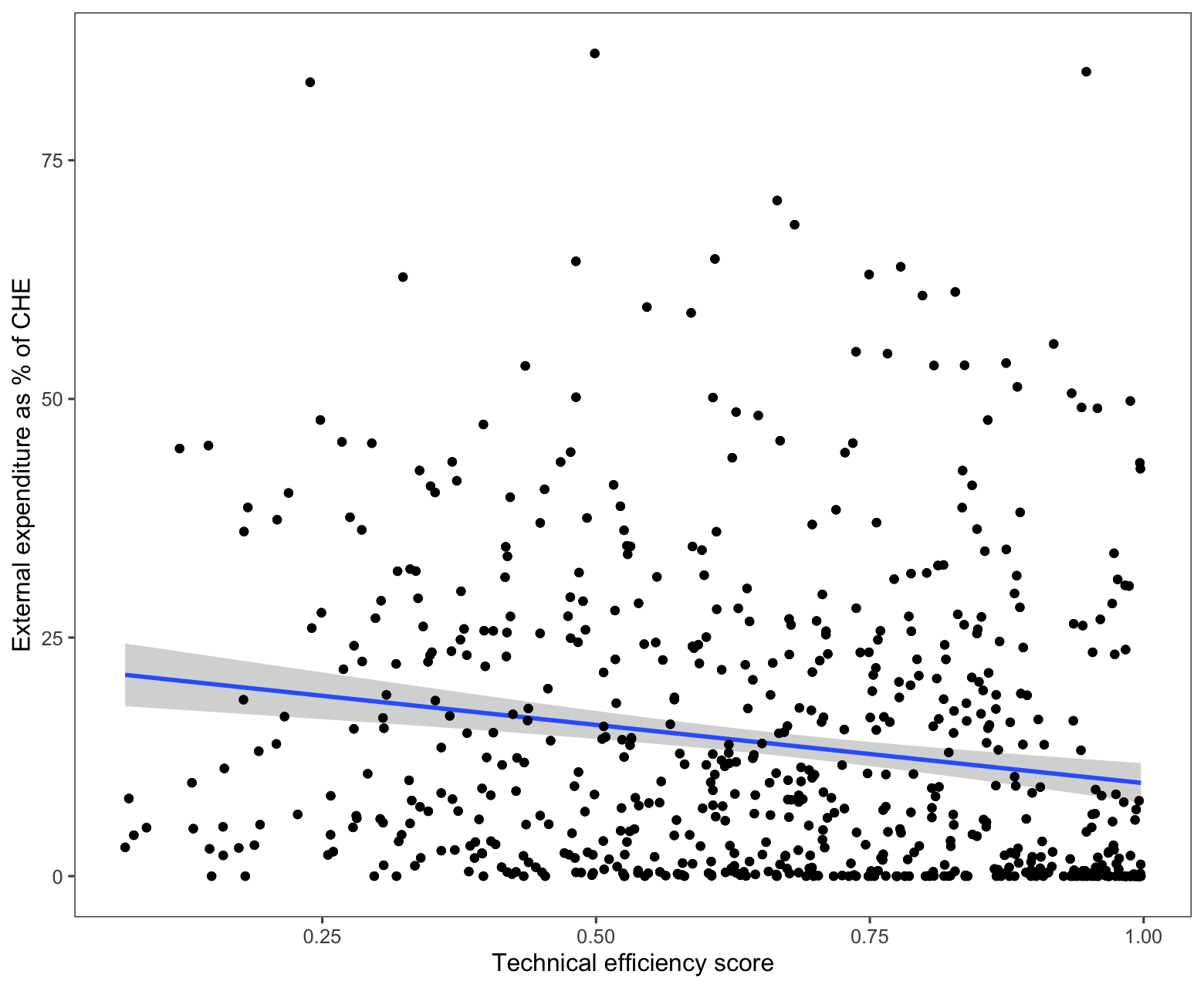
**

*Notes:* blue solid line stands for a linear unadjusted fit between the two variables, whereas greyish shadowed areas are for 95% CI intervals. Black circles are for the observations. CHE: Catastrophic Health Expenditure, HDI: Human Development Index, GNI: Gross National Income, GS: Government spending, OOPE: Out-of-pocket expenditure, USD: United States Dollars, DAHS: Development assistance for health spending.

**Table G.** Non-imputed results of the robust coefficients in the truncated regression for the reciprocal of the efficiency score (N=64 countries, n=448 observations)

| VARIABLES | | | COEFF | SE | P-value |
| --- | --- | --- | --- | --- | --- |
| Intercept |  |  | 4.742 | 3.81 | 0.215 |
| Rule of Law |  |  | -1.581 | 0.98 | 0.106 |
| Antenatal Care Coverage | |  | 0.025 | 0.03 | 0.486 |
| GNI per capita in USD | |  | 0.001 | 0.00 | 0.004 |
| CHE as % of GDP | |  | -0.170 | 0.23 | 0.463 |
| CHE per capita in USD | |  | -0.012 | 0.01 | 0.073 |
| Population per KM^2^ | |  | -0.013 | 0.01 | 0.039 |
| Human Development Index (HDI) | | | -23.839 | 10.16 | 0.019 |
| HIV prevalence | |  | -0.774 | 0.30 | 0.009 |
| OOPE as % of the total HIV spending | | | 0.130 | 0.05 | 0.017 |
| DAHS per total HIV spending ratio | | | 0.053 | 0.03 | 0.077 |

*Notes*: CHE: Catastrophic Health Expenditure, HDI: Human Development Index, GNI: Gross National Income, OOPE: Out-of-pocket expenditure, USD: United States Dollars, DAHS: Development assistance for health spending

**Table H.** Sensitivity analysis II for the reciprocal of the efficiency score

| **[a]: Sensitivity analysis estimates**  Variables/Models | | **Model C** | **Model D** | **Model E** | **Model F** | **Model G** |  |
| --- | --- | --- | --- | --- | --- | --- | --- |
|  |  | - bottom 5% outputs | - upper 5% outputs | - bottom 5% inputs | - upper 5% inputs | - bottom/upper 5% inputs/outputs |  |
|  |  |  |  |  |  |  |  |
|  |  | Coeff | Coeff | Coeff | Coeff | Coeff |  |
| (Intercept) |  | 10.113 | -7.310 | 0.827 | 2.012 | 4.958 |  |
| Rule of Law |  | -4.829 | -41.155 | -54.169 | -58.982 | -4.640 |  |
| Antenatal Care Coverage | | 0.021 | -0.409 | -0.793 | -0.831 | 0.031 |  |
| GNI per capita in USD | | 0.002 | 0.007 | 0.010 | 0.009 | 0.001 |  |
| CHE as % of GDP | | -0.244 | 0.308 | 0.216 | 0.128 | -0.042 |  |
| CHE per capita in USD | | -0.029 | -0.094 | -0.144 | -0.146 | -0.026 |  |
| Population per KM^2^ | | -0.030 | 0.062 | 0.063 | 0.076 | -0.024 |  |
| Human Development Index (HDI) | | -0.514 | -1.832 | -2.264 | -2.262 | -0.451 |  |
| HIV prevalence | | -1.694 | -9.102 | -14.106 | -12.599 | -1.198 |  |
| OOPE as % of the total HIV spending | | 0.206 | 0.764 | 1.043 | 0.945 | 0.184 |  |
| DAHS per total HIV spending ratio | | 0.062 | 0.134 | 0.163 | 0.053 | 0.065 |  |
| Number of observations (countries) | | 643 (76) | 619 (77) | 659 (77) | 617 (74) | 571 (72) |  |

Notes: Base model without any outlier for spending in HIV/AIDs and outcomes (ART) + PMTCT. OOPE: Out-Of-Pocket Expenditure. DAHS: Development assistance for health spending. ^a^ Sample schematic can be found in Figure 1. 95% CI and further information on the models upon request.

| **[b]: Summary predicted values** | Average DEA  score | Average  95% CI | | T-test specification ^b^ | T-test  (p-value) |
| --- | --- | --- | --- | --- | --- |
| (Model A) | 0.671 | 0.653 | 0.689 | (BM VS A) | 0.946 |
| (Model B) | 0.671 | 0.653 | 0.688 | (BM VS B) | 0.963 |
| (Model A+B) | 0.670 | 0.653 | 0.688 | (BM VS A+B) | 0.969 |
| (Model C) | 0.679 | 0.653 | 0.688 | (BM VS C) | 0.469 |
| (Model D) | 0.676 | 0.662 | 0.697 | (BM VS D) | 0.651 |
| (Model E) | 0.670 | 0.658 | 0.693 | (BM VS E) | 0.983 |
| (Model F) | 0.657 | 0.652 | 0.687 | (BM VS F) | 0.337 |
| (Model G) | 0.676 | 0.639 | 0.676 | (BM VS G) | 0.642 |

*Notes:* t-tests were computed using two-tails and alpha=0.05. ^b^ Mean differences were computed between Model numbers detailed within the brackets. BM stands for Base Model.

**Figure B.** Histogram of efficiency scores from the base model

**Figure C.** Predicted efficiency scores by country from the base model (N=78 countries)

*Notes:* countries without boxes only have one single data-point. DEA scores stands for technical efficiency scores.

**Figure D**. Distribution of our main outputs and input


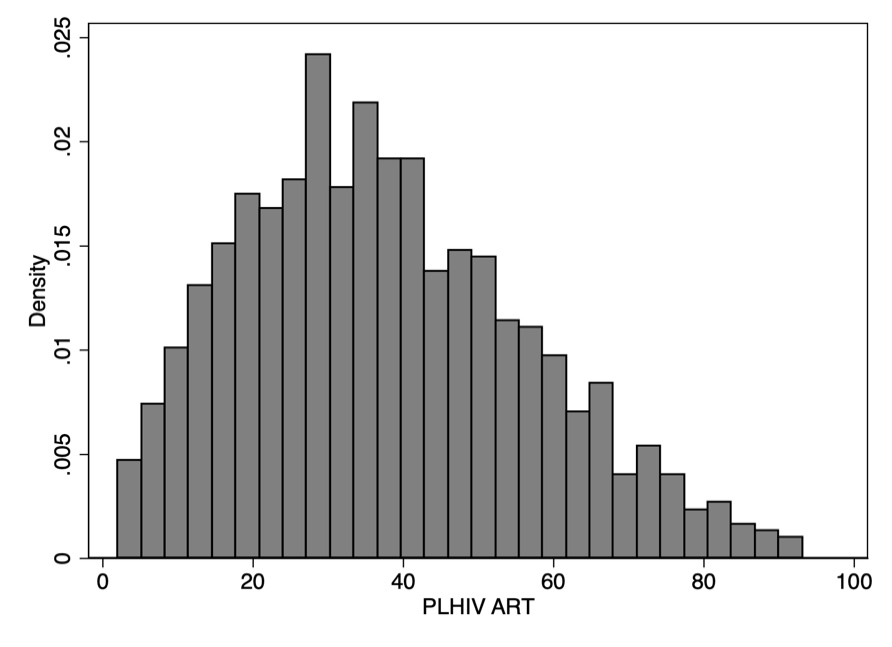


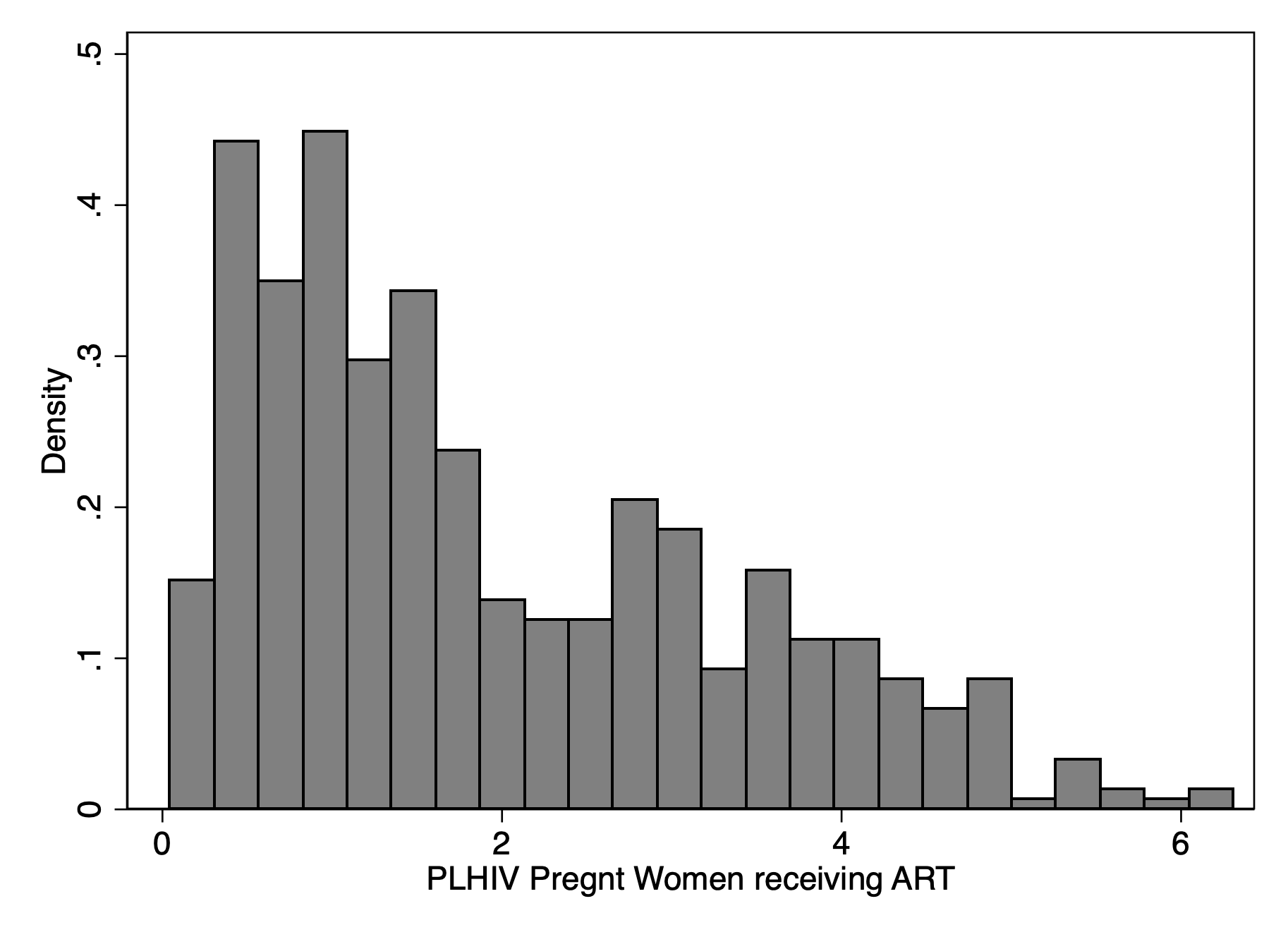


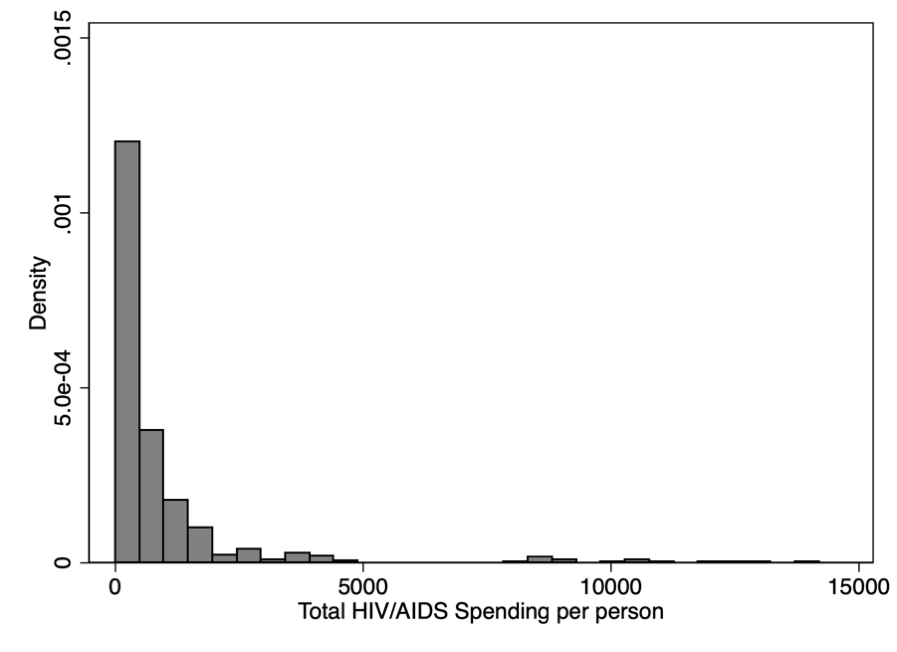


**Figure E.** Average bias-corrected efficiency scores by UNAIDS region

*3.* Values were extracted from the base model.

**Figure F.** Scatterplot matrix between the estimated efficiency scores and input and outputs


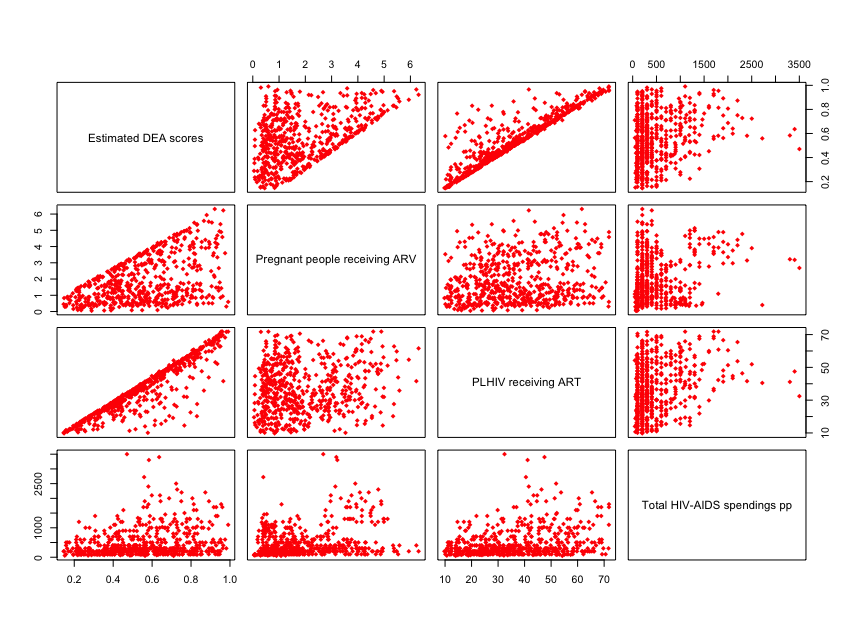


*Notes:* Scatterplot based on the variables used for the main model (Table 4). pp: per capita. ART: antiretroviral therapy. ARV: antiretroviral. PLHIV: people living with HIV.

**Figure G.** Scatterplot matrix between the estimated efficiency scores and independent variables


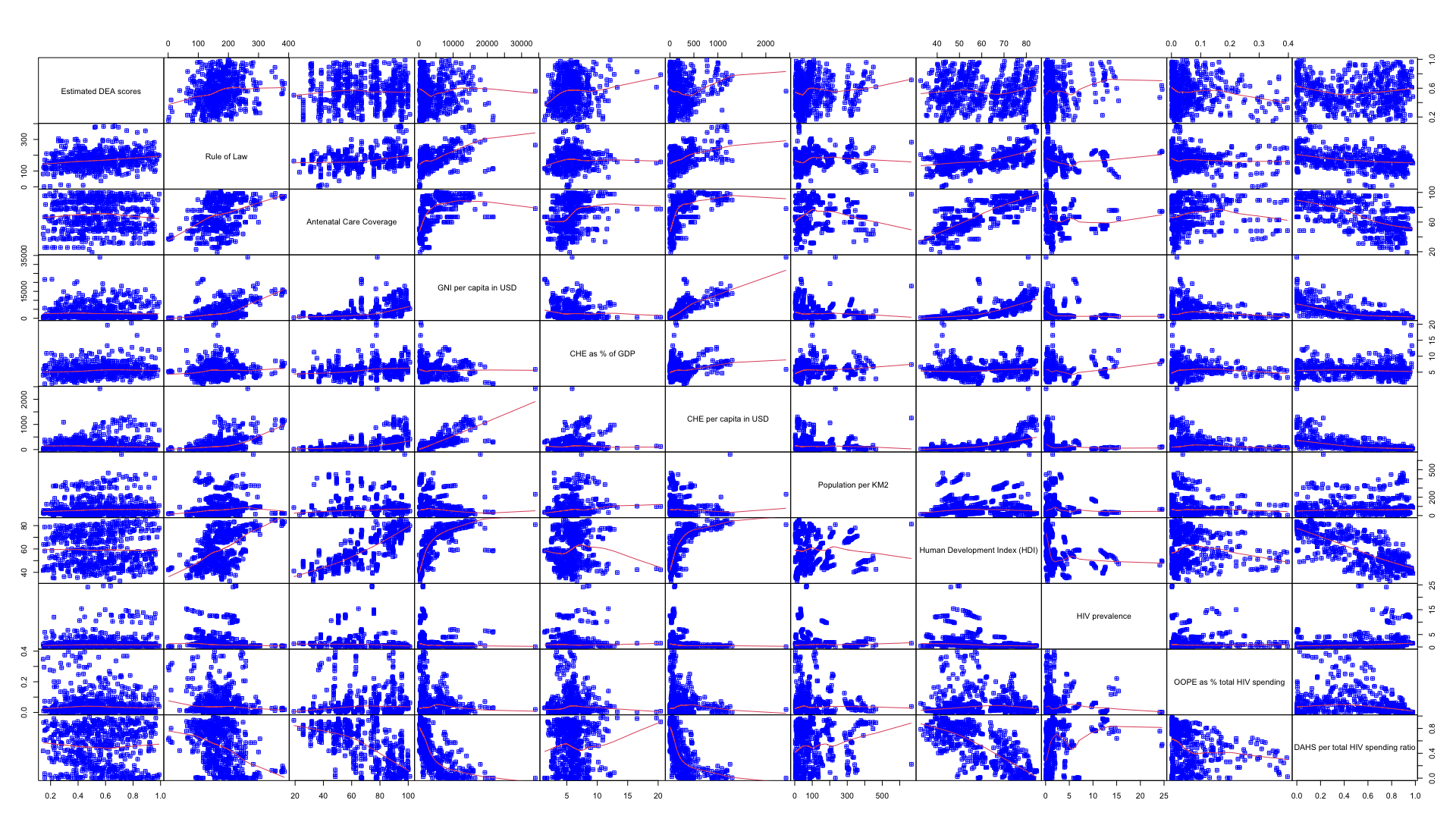


*Notes:* Scatterplot based on the variables used for the main model (Table 4). pp: per capita. Red lines stand for polynomial fit between variables.

**References**

1. Cooper WW, Seiford LM, Zhu J. Data envelopment analysis: History, models, and interpretations. Handbook on data envelopment analysis: Springer; 2011. p. 1-39.

2. Charnes A, Cooper W, Lewin AY, Seiford LM. Data envelopment analysis theory, methodology and applications. Journal of the Operational Research society. 1997;48(3):332-3.

3. Simar L, Wilson PW. Two-stage DEA: caveat emptor. Journal of Productivity Analysis. 2011;36(2):205.

4. Simar L, Wilson PW. Estimation and inference in two-stage, semi-parametric models of production processes. Journal of econometrics. 2007;136(1):31-64.

5. Badunenko O, Tauchmann H. Simar and Wilson two-stage efficiency analysis for Stata. The Stata Journal. 2019;19(4):950-88.

6. Royston P. Multiple imputation of missing values. The Stata Journal. 2004;4(3):227-41.
