# Supplementary material for: Technical efficiency of national HIV/AIDS spending in 78 countries between 2010 and 2018: A data envelopment analysis
Source: PLOS Glob Public Health. 2022 Aug 1;2(8):e0000463. doi: 10.1371/journal.pgph.0000463 (PMC10022340; doi:10.1371/journal.pgph.0000463)
Supplement: Attachment — Submitted filename: Response to editors points.docx [file pgph.0000463.s004.docx]

**Thank you very much for the additional guidance below. We have now addressed the points provided but please let us know if we missed or misunderstood anything.**

1. Please provide separate figure files in .tif or .eps format only and ensure that all files are under our size limit of 20MB.

**RESPONSE: Done, all uploaded files are under 20MB and are in .tif format.**

2. Your co-authors:
Kasim Allel -kasim.allel1@student.lshtm.ac.uk
Jolene Skordis -j.skordis@ucl.ac.uk
Hassan Haghparast-Bidgoli -h.haghparast-bidgoli@ucl.ac.uk
,have not confirmed authorship of the manuscript. We have resent them the authorship confirmation email; however please check that the above email address for them is correct and follow up personally to ensure they confirm.

Please note that we cannot proceed your manuscript  until we have received confirmations from all co-authors.

**RESPONSE: Thank you very much for flagging this. The authors listed above should have confirmed their authorship. Please do let us know if not.**

3. Please note that your Data Availability Statement is currently missing direct links to access each database. If your manuscript is accepted for publication, you will be asked to provide these details on a very short timeline. We therefore suggest that you provide this information now, though we will not hold up the peer review process if you are unable.

**RESPONSE: Done**

4. Please amend your Financial Disclosure statement. If you did not receive any funding for this study, please simply state: “The authors received no specific funding for this work.”

**RESPONSE: Done**

5. Please update your Competing Interests statement. If you have no competing interests to declare, please state: “The authors have declared that no competing interests exist.”

**RESPONSE: Done**

6. We have noticed that you have uploaded supporting information but you have not included a list of legends.  Please add a full list of legends for all supporting information files (including figures, table and data files) after the references list.

**RESPONSE: Done. A list of legends is now included in both the main manuscript and supplement.**
